# Meiotic maps of sockeye salmon derived from massively parallel DNA sequencing

**DOI:** 10.1186/1471-2164-13-521

**Published:** 2012-10-03

**Authors:** Meredith V Everett, Michael R Miller, James E Seeb

**Affiliations:** 1 School of Aquatic and Fishery Sciences, University of Washington, Box 355020, Seattle, WA 98195-5020, USA; 2 Institute of Molecular Biology and Institute of Neuroscience, 1229 University of Oregon, Eugene, OR 97403-1229, USA

**Keywords:** RAD sequencing, Meiotic map, Linkage map, SNP

## Abstract

**Background:**

Meiotic maps are a key tool for comparative genomics and association mapping studies. Next-generation sequencing and genotyping by sequencing are speeding the processes of SNP discovery and the development of new genetic tools, including meiotic maps for numerous species. Currently there are limited genetic resources for sockeye salmon, *Oncorhynchus nerka*. We develop the first dense meiotic map for sockeye salmon using a combination of novel SNPs found in restriction site associated DNA (RAD tags) and SNPs available from existing expressed sequence tag (EST) based assays.

**Results:**

We discovered and genotyped putative SNPs in 3,430 RAD tags. We removed paralogous sequence variants leaving 1,672 SNPs; these were combined with 53 EST-based SNP genotypes for linkage mapping. The map contained 29 male and female linkage groups, consistent with the haploid chromosome number expected for sockeye salmon. The female map contains 1,057 loci spanning 4,896 cM, and the male map contains 1,118 loci spanning 4,220 cM. Regions of conservation with rainbow trout and synteny between the RAD based rainbow trout map and the sockeye salmon map were established.

**Conclusions:**

Using RAD sequencing and EST-based SNP assays we successfully generated the first high density linkage map for sockeye salmon.

## Background

Meiotic maps are a critical component of the process of unraveling the complexities of the genomics of duplicated taxa [1]. Whole genome duplication with concomitant re-diploidization is broadly acknowledged as the major driving force in adaptation and speciation [2-4]. Positive selection is enriched in duplicated genes [5,6]. Sorting the relationships among orthologs, homologs, and homeologs in duplicated genomes can be both daunting [1] and rewarding [7]. Meiotic maps have been used to unravel the interactions among linkage, pseudolinkage, homeologous pairing, and interference in duplicated teleosts for more than three decades [7-9]. More recently, meiotic maps have become important for comparative genomics [10] and association mapping studies [11].

Techniques that associate genotypes with phenotypes, whether disease, physiological, or ecological traits, can improve our understanding of local adaptation [12]. Understanding how organisms interact with their local environments is increasingly important for conservation and management of natural populations. In order to understand these relationships, studies are turning to association-mapping techniques where thousands of ordered markers across a genome are scanned for association with a trait. The most powerful analyses require thousands of markers in a known order, as found in a genome sequence or high-density linkage map. Such tools are currently unavailable for most non-model organisms, though the rapid advance of high throughput sequencing techniques is facilitating their creation [12,13].

The emergence of genotyping by sequencing (GBS), [14,15] provides exciting opportunities to rapidly establish meiotic maps for non-model species. Previously, marker discovery posed a major hurdle to the development of high density linkage maps. Early studies generally included the mapping of only a few hundred markers [16]. GBS now enables thousands of SNPs to be genotyped rapidly at relatively low cost [17,18]. As a result, dense mapping studies are set to become common in wild populations [19], providing opportunities for association mapping, genome scans, and comparative mapping [1].

Salmonids with their residual polyploidy provide an excellent opportunity for study. Salmonids are an iconic family of fish, with both cultural and economic value, that naturally inhabit the North Pacific and North Atlantic Oceans. To date no published genome sequence for salmonids exists, though there are extensive SNP and EST resources for a few species e.g., [20]. Dense linkage maps, containing thousands of markers, currently only exist for rainbow trout *Oncorhynchus mykiss*[11,16] and Atlantic salmon *Salmo salar* reviewed in [21,22]; these species are intensively cultured for both food and sport fishing on several continents. The ongoing development of linkage maps in additional salmonid species has provided key insights into adaptation, sex determination, disease resistance, and other factors important to both conservation of wild populations and aquaculture. These maps also offer opportunities for comparative genomics [10,15,23-25]. A collateral benefit is that converting batteries of mapped SNPs to high-throughput genotyping assays will greatly enhance population genetic studies and genome scans that are becoming a backbone of conservation genomics [26].

A salmonid of special interest is the sockeye salmon *O. nerka* which is culturally important and supports some of the most valuable commercial fisheries by coastal nations throughout the subarctic North Pacific Ocean [27,28]. Genomic resources are scant; no genetic maps exist for sockeye salmon. Although populations are robust in the northern climes, some cornerstone populations in Canada and the USA are threatened or endangered [29,30]. As climate change intensifies, concerns intensify that thermal [31] or disease [32] challenges or both [33,34] will further threaten commercial and subsistence economies [35]. This threat, occurring during an era empowered by the advent of conservation genomics [12], precipitates a need for genomic resources to enable studies of genetic diversity, adaptive variation, and genotype-by-environmental interactions in sockeye salmon. Additionally, sockeye salmon exhibit a variety of unique life histories [28,36], and additional genomic resources are necessary for determining the genetic basis for these diverse life history traits.

Our study had two goals. First was to discover thousands of novel SNPs in sockeye salmon using restriction site associated DNA sequencing (RAD) tags, [37-39]. Our second goal was to use these markers to generate a high density linkage map, with paired-end sequences [40] used to expand template length to annotate as many SNPs as possible. We also incorporated EST-based SNPs, available through existing 5’-nuclease assays, into our genetic map because the substantial majority of RAD tags reside in non-coding sequences. We successfully discovered putative SNP-containing loci using RAD tag sequencing in a single family and used these to construct male and female consensus maps. The female map contains 1,057 markers and the male map contains 1,121 markers.

## Results

### SNP discovery in parents

We had 13 single-pair matings available for this study (Table 1).

**Table 1:** Experimental matings for SNP genotyping

**Family**	**Number of individuals**	**Genotyping method**	**Segregating EST loci ineach family**
HX1	45	5’-nuclease	44
HX2	45	5’-nuclease	47
HX3	45	5’-nuclease	52
HX5	45	5’-nuclease	53
HX6	93	5’-nuclease	57
HX7	45	5’-nuclease	48
HX8	45	5’-nuclease	52
HX9	45	5’-nuclease	54
HX10	45	5’-nuclease	50
HX11	45	5’-nuclease	52
HX12	45	5’-nuclease	56
HX13*	45	5’-nuclease	53
HX13-WL*	96	RAD sequencing, 5’-nuclease	53
HX14	45	5’-nuclease	49
HX-Parents†	6	RAD sequencing, 5’-nuclease	100

Number of progeny included from each family, the genotyping method used on each, and the number of segregating EST loci.

* HX13 and HX13-WL were two different sets of offspring from the same parents. †Parents were the male and female from families HX6, HX8, and HX13. The additional parents were used for the assembly of paired end contigs.

**Figure 1:**
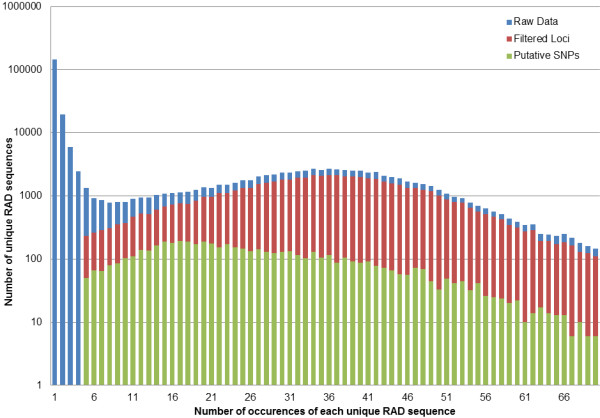
**Frequency distribution of unique RAD tag sequences from the parents of family HX13-WL before and after filtering and SNP discovery.** Blue bars are the number of occurrences of each raw RAD sequence in both the male and female. The peak at the left is formed by sequences that only occur between one and five times and were removed from further analysis during filtering. Red bars show the distribution of the shared loci identified between the two parents (Table 1). Green shows the distribution of the 3,430 putative SNP containing loci from both parents. Note that the bars are not cumulative. The leftward shift in the polymorphic sequence distribution reflects that these loci were heterozygous in one or both parents and therefore have lower counts. There are scattered occurrences above a frequency count of 70, but the tail quickly asymptotes to near 0 with scattered single peaks.

**Figure 2:**
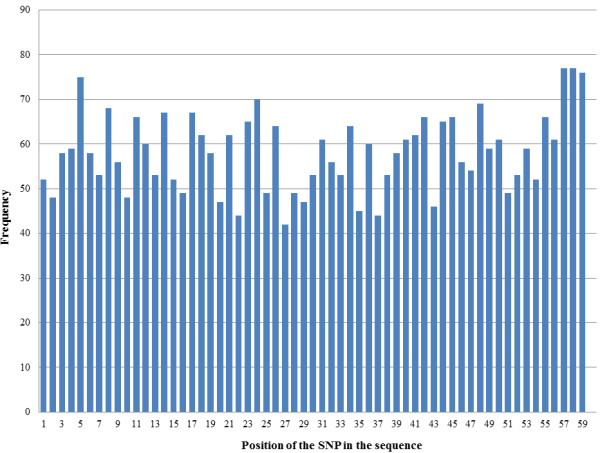
**The frequency distribution of the position of each SNP in the RAD tag sequences discovered in the HX13-WL parents.** After removal of the restriction site and barcode, and trimming the 3’end bases, 59 nucleotides remain. If putative SNPs were due to sequencing error, a significant enrichment of SNPs near the 3’ end of the RAD tag sequences would be expected. While a small peak in the 3’-end remains, the majority of bias has been removed and later data filtering not did not remove a larger percentage of SNPs from these locations.

The parents from a single family, HX13-WL, were selected for SNP discovery. Over 3.5 million RAD tag sequences were generated in both the male and female parents (Table 2). These RAD tag sequences as well as the RAD tag sequences from the offspring (see below) were deposited in the NCBI short read archive (Accession: SRA051991.1). These sequences were grouped and counted resulting in more than 250,000 unique sequences in each parent (Table 2). A frequency histogram of the number of occurrences per unique sequence (Figure 1) shows a sharp peak of sequences that only occur between one and five times in either parent. We filtered these low count sequences and grouped those remaining into loci shared between the two parents (see methods). This resulted in a set of 64,613 shared loci (Figure 1, Table 2) that contain both monomorphic and polymorphic RAD tags. Of these, 61,183 loci were monomorphic, 120 loci were homozygous in each parent but polymorphic between the parents, 1,596 loci were polymorphic in one parent and homozygous in the other and 1,714 were polymorphic in both parents. Only loci that were polymorphic within one or both parents could be mapped, resulting in 3,430 putative SNPs to be analyzed further (Table 2, Figure 1). The location of each SNP in the RAD tag was relatively evenly distributed (Figure 2), indicating that the SNPs were unlikely to represent sequencing errors due to drop off of base quality near the end of sequence reads [11].

**Table 2:** RAD tag and SNP discovery in the parents from family HX13-WL

**Individual**	**Filtered reads**	**Unique sequences**	**Shared loci**	**Putative SNPs**
HX13 Female	3,995,897	265,492	64,613	3,430
HX13 Male	3,517,798	258,051	64,613	3,430

Filtered reads is the number of sequence reads after each sequence was trimmed to 70 bp; sequences with less than 80% chance of being error free were discarded from further analysis. Unique sequences is the number of unique RAD tag sequences found in the data from each parent using Perl scripts. Shared loci is the number of unique loci, shared between the two parents; this includes both polymorphic and monomorphic loci. Putative SNPs is the SNP containing loci from the shared loci.

### Genotyping of offspring

**Figure 3:**
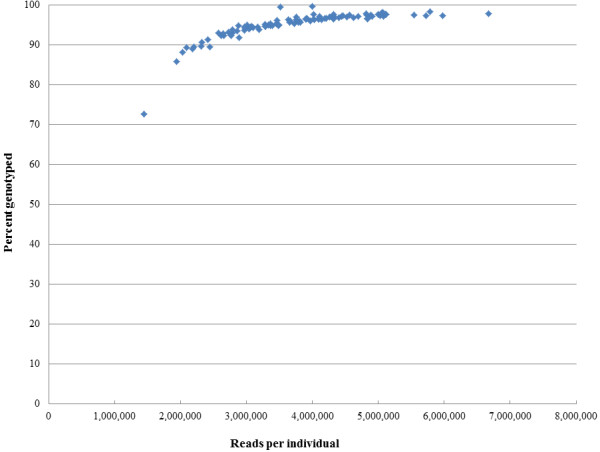
**The percentage of markers genotyped successfully in each individual offspring with GBS versus the number of quality filtered reads in each individual.** The number of reads was calculated from merged data of the two sequencing runs. Eighty-eight out of 96 offspring were successfully genotyped at over 90 percent of the loci, and all individuals were included in linkage analysis.

Sequencing of RAD tags was carried out on 96 offspring from family HX13-WL (Table 1, Table 3). Two rounds of sequencing were necessary to achieve sufficient depth of coverage to accurately call genotypes in the offspring. The first round was carried out on the Illumina GAII, and the second round on the Illumina HiSeq 2000 (Table 3). The combined sequencing efforts resulted in between 1,443,900 and 6,672,291 filtered reads per individual with a mean value of 3,754,867 ± 1,007,970. RAD tags from each offspring were aligned to the 3,430 putative SNPs scored in the parents, and coverage counts for each allele were calculated. Coverage values ranged from 0 to 2,246 reads with an average of 49.0 aligned reads. After converting aligned read counts to genotype calls, the majority of offspring (88/96) were successfully genotyped at over 90% of the putative SNPs discovered in the parents (Figure 3).

**Table 3:** Pooling strategy for Illumina sequencing

**Illumina sequencing technology**	**Number of individuals perlane**	**Number of lanes**
GAII paired end	6 parents	1
GAII single end	16 progeny	6
HiSeq 2000 single end	24 progeny	3

Parents were sequenced with the GAII (paired end). Offspring were first sequenced with the GAII (single end); the depth of coverage was insufficient for genotyping and a second round of sequencing was done using the HiSeq 2000 (single end). The results of the GAII single end and HiSeq 2000 single end runs were filtered and trimmed to the same lengths and combined for analysis.

In addition to RAD-based SNPs, we genotyped EST-based SNPs using 127 existing 5’-nuclease assays on parents and 45–141 offspring from each of the thirteen families (Table 1). After filtering, each family contained between 44 and 57 segregating loci, with a total of 100 loci segregating across all families (Table 2). Linkage relationships in these families were merged into the final map.

Both RAD and EST genotypes between parents and offspring were compared for inheritance error (genotypes in the offspring that do not correspond to the parental types), and erroneous genotypes were converted to missing data. Next the genotypes were screened for segregation distortion. The salmonid genome contains an ancient whole genome duplication and consequently contains many duplicate regions [41]. The presence of these duplications has resulted in high false positive rates in previous SNP discovery efforts. In our data set, markers with significant segregation distortion (χ^2^ test, p < 0.05, deviation from 1:1 or 1:2:1 segregation patterns) may represent these paralogous sequence variants (PSVs), true distortion due to other genomic factors, or genotyping errors. Our genotyping method currently does not allow identification of genotyping error versus true distortion, thus we exclude all markers with significant distortion. Out of 3,530 putative SNPs, 1,758 (49%) showed significant segregation distortion and were removed from further analysis. The majority of these loci showed allelic distributions that indicated that they were likely PSVs (majority of all individuals heterozygous). All of the loci removed for segregation distortion were from the RAD tag sequences. Finally, after removing markers with greater than 25% missing data, 1,725 RAD and 5’-nuclease based SNPs were used in linkage analyses.

### Meiotic map construction

Linkage mapping was carried out in two steps. First, the genotypes from the 53 EST loci segregating in family HX13-WL were combined with the RAD tag genotypes, and mapping was carried out on the combined RAD-EST dataset from family HX13-WL. Second, mapping was done on the EST loci that were segregating in the remaining families to maximize the number of ESTs that could be included in the final map (Table 1). Linkage groups identified among these EST loci were merged with the HX13-WL linkage maps to create male and female consensus maps.

The female RAD-EST map from family HX13-WL contained 1,050 markers distributed among 29 linkage groups, consistent with the expected number of haploid chromosomes for sockeye salmon [42]. The total map length was 4,943.7 cM. Twenty three markers failed to map and were excluded from further analysis. Additionally, this map contained two small fragments, each containing only two markers, which were discarded from further analysis.

The male RAD-EST map initially contained 28 linkage groups and a total length of 4,352.8 cM. Visual inspection of individual linkage groups and comparison to the female map revealed that one male linkage group corresponded to three female linkage groups (female linkage groups LG27, LG28, and LG29). Inspection of the recombination frequency matrix for the corresponding male and female linkage groups revealed that the male linkage group contained two linkage groups joined through an apparently spurious linkage relationship due to a single marker. No corresponding relationship could be observed in the female. This marker was removed, and the two linkage groups were separated. One of the divided male linkage groups matched to female LG28 and the other matched to the remaining two female groups (LG27, 29). Additionally, one female linkage group corresponded to two male linkage groups, LG7a and LG7b. LG7a contained only five markers and may represent a fragment of the larger LG7b. A number of markers within LG7a had LOD >4 with markers in 7b, suggesting that these are part of a single group. After corrections, the male RAD-EST linkage map contained 1,112 markers distributed among 29 linkage groups and eight small fragments containing only two or three markers. These fragments were discarded from further analysis. Sixteen markers failed to map. The male map length was 4,310.3 cM.

There were 29–35 segregating loci per parent in the EST data set from the additional families (Table 1). As a result, only small linkage groups were established, consisting of between two and four markers per group with up to nine groups per family. Overlaps of two or more markers between groups among the families allowed some groups to be merged. After merging, 31 linkage relationships in the males and 28 in the females, consisting of between two and five markers each, were established. These fragments were compared to the RAD-based map from family HX13-WL and merged where two or more markers overlapped. In this fashion, thirteen additional EST loci were added to the consensus map, seven additional markers in the female map and six additional markers in the male. The remaining EST groups were excluded from further analysis.

**Figure 4:**
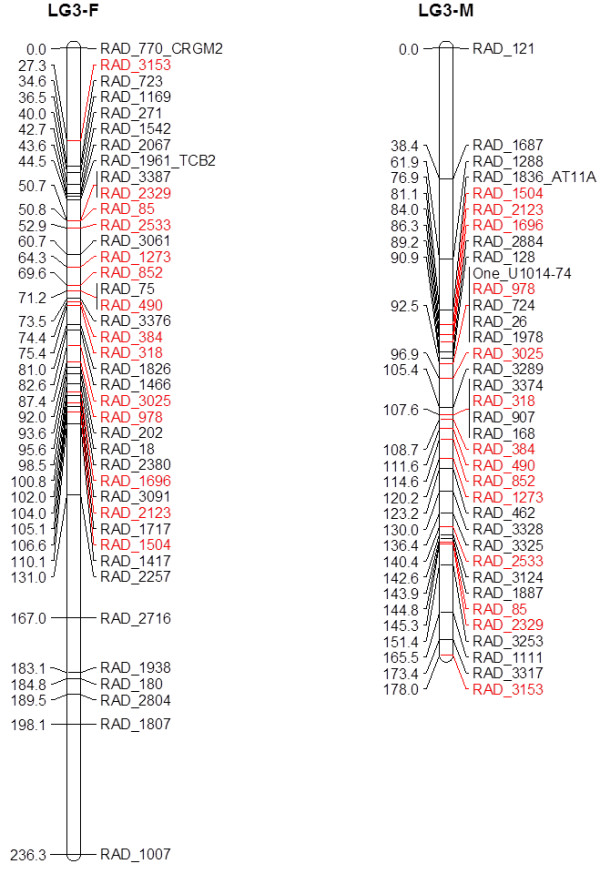
**Female (F) and Male (M) consensus maps for linkage group LG2.** Markers shared between the male and female maps are in red. Marker names beginning with RAD_ are rad tag SNPs and names beginning with One_ are from 5’-nuclease assays.

Merging the EST markers did not alter the number of linkage groups. The consensus female map contained 1,057 markers in 29 linkage groups and a total length of 4,895.8 cM and the consensus male map contained 1,118 markers with a total length of 4,220.0 cM across 29 linkage groups (Figure 4, Additional files 1, 2, 3, 4, 5, 6 and 7).

There were 457 overlapping loci between the male and the female maps (40% of total female markers and 41% of total male markers) (Figure 4, Additional files 1, 2, 3, 4, 5, 6 and 7). In all cases the overlapping loci were assigned to the same linkage groups. The male map had two linkage groups (LG7a and LG7b) that corresponded to a single linkage group (LG7) in the female map, resulting in 28 unique male groups when compared to the female map. Examination of the recombination matrix for the corresponding female linkage group did not support splitting this group. Additionally, as noted above, male LG27 corresponds to female LG27 and LG29.

A number of linkage groups contain minor order discrepancies between the male and the female maps (Additional files 1, 2, 3, 4, 5, 6 and 7). The markers contained these groups consistently in even when the LOD score is varied. Removal of each marker and repositioning using try.seq in both the male and the female was unable to resolve the order variation in these groups. In no cases did shared markers map to different linkage groups between the male and the female. Linkage groups containing marker order discrepancies include LG14, LG20, LG22, and LG27.

**Figure 5:**
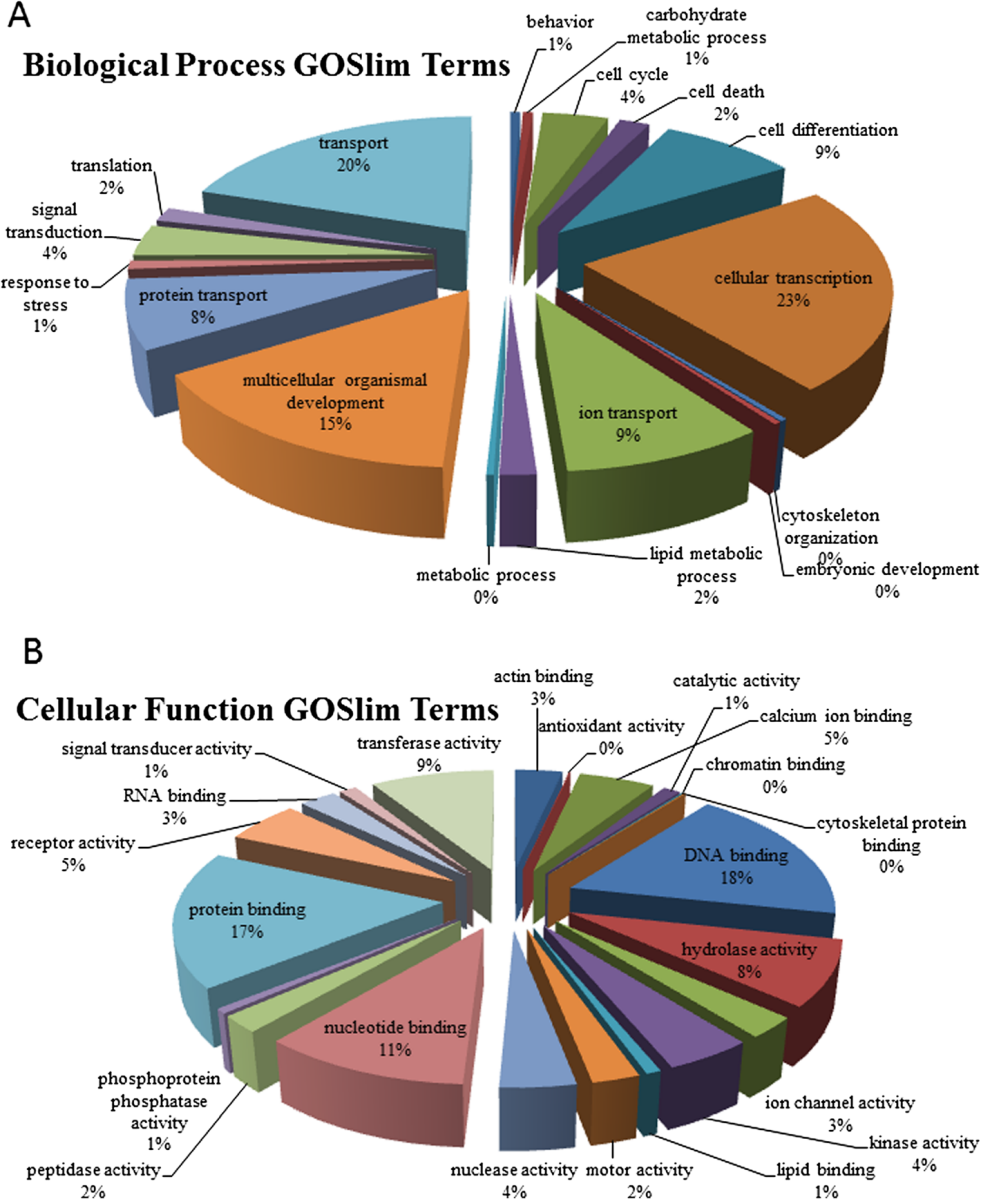
**Recombination matrices for A) male and B) female LG3.** Each is a heat map with recombination fraction below the diagonal and corresponding LOD score above the diagonal between all pairwise combinations of markers in LG3. Above the diagonal warmer colors (oranges, reds) are higher LOD scores with the highest scores being the darkest reds. Below the diagonal are recombination fractions with lower values in warmer colors. Note that majority of high values occur closer to the diagonal in the female and that the female has more recombination (higher recombination fractions in blue) overall.

Recombination in the male was reduced over what was observed in the female. The male map contained 61 more markers than the female but was 675.8 cM shorter overall. As not all markers are shared between the male and female, it is inappropriate to make a direct comparison between corresponding linkage groups. Rather, we compared the distance between the most proximal and distal shared pair within each group. The ratio for individual linkage groups ranged from 0.71:1 to 1.71:1. Additionally, the male had more extensive clusters of non-recombinant markers across linkage groups (Table 4, Figure 4, Figure 5 Additional files 1, 2, 3, 4, 5, 6 and 7).

**Table 4:** Recombination ratios among individual male and female linkage groups

**Linkage Group**	**Female cM**	**Male cM**	**Female: Male**
1	42.7	25	1.708
2	119.5	123	0.97154
3	79.3	96.9	0.81837
4	153.5	160.7	0.9552
5	72	76.6	0.93995
6	91.3	72.8	1.25412
7a	6.9	6.3	1.09524
7b	57.1	57.6	0.99132
8	71.2	81.3	0.87577
9	197.4	224.1	0.88086
10	155.4	126.2	1.23138
11	98.8	99.5	0.99296
12	113.2	123	0.92033
13	144.4	131.4	1.09893
14	126.9	138.9	0.91361
15	151.3	120.2	1.25874
16	80.9	84.1	0.96195
17	39.8	44.3	0.89842
18	120.9	115	1.0513
19	51.5	50.5	1.0198
20	156.8	212.8	0.73684
21	72.1	77.8	0.92674
22	189	176.2	1.07264
23	113.1	159.6	0.70865
24	104.2	115.9	0.89905
25	76.9	74.5	1.03221
26	53.3	46.8	1.13889
27	90.7	82.4	1.10073
28	71.3	91.3	0.78094
29	22.7	22.2	1.02252
Total	2924.1	3016.9	0.96924

The distance between the most proximal and distal of the shared markers was calculated for the male and female on each linkage group and a ratio was compared from these distances. Male LG27 corresponds to female LG27 and LG29; the distances between the shared markers from LG27-F and LG29-F are both included. LG7 is split in the male and 7a and 7b are the shared markers from each of the male linkage groups.

### Comparison to rainbow trout

The complete set of 64,613 putative RAD tag loci from sockeye salmon (Table 2) was compared to 40,649 RAD tag loci from rainbow trout [11]. A BLASTN comparison revealed 196,021 pairwise BLAST hits between the two species with an e-value of less than 10^-4^. Many of these hits, however, were matches for only a portion of a tag or contained gaps (e.g. 28 out of 59 nucleotides in the tag match). After filtering (see methods), 16,989 full length matches between the species were discovered. These 16,989 matches contain monomorphic RAD tag sequences and both mapped and unmapped SNPs from both species. We filtered the 16,989 matches for those contained in each linkage map to examine synteny between the two species. Fifty-five markers were shared between the 1,718 SNPs included in the sockeye salmon linkage maps and the 4,888 SNPs in the rainbow trout linkage map [11]. The 55 markers were not evenly distributed across the sockeye linkage map (Table 5). Seven sockeye salmon linkage groups contained no hits to the mapped rainbow trout RAD tags. Among the remaining 22 linkage groups, the number of shared tags between groups ranged from a single tag to four. Five sockeye linkage groups contained matches to two separate rainbow trout linkage groups. Two rainbow trout linkage groups matched to two sockeye linkage groups, with all remaining matches between single linkage groups.

**Table 5:** **Comparison between rainbow trout and sockeye salmon linkage groups **[ [11]]

**Sockeye salmon linkage group**	**Rainbow trout linkage group**	**Number of hits**
1	-	-
2	WS25	2
3	WS25	1
4	WS3, WS19	2,2
5	WS24	1
6	WS12	1
7	WS10	3
8	WS26	1
9	WS5, WS18	1,1
10	-	-
11	WS16	1
12	WS4	2
13	WS7	3
14	WS17, WS18	1,1
15	-	-
16	-	-
17	WS1	2
18	WS22	1
19	WS11	2
20	WS8, WS9	2,1
21	WS29	3
22	WS21	2
23	-	-
24	WS3, WS5	2,2
25	WS20	2
26	-	-
27	WS13	2
28	WS27	2
29	-	-

In cases where there are hits to more than one linkage group, the number of hits for each is listed in the same order as the linkage groups. Two rainbow trout linkage groups, WS25 and WS18, match to two separate sockeye salmon linkage groups. The WS designations are the names from Miller et al. [11], 1,2,3,4 and 5 references the specific *O. mykiss* chromosomes.

### Paired-end sequencing

Paired-end sequencing of genomic DNA from six parents, to provide longer contigs for annotation, produced between 3,517,798 and 3,995,897 filtered reads per individual for assembly. Out of 6,860 potential contigs, 5,722 were successfully assembled with a minimum length of 150 bp. Average contig length was 211 ± 30 bp, with an average coverage value of 38.25 ± 33.76 reads.

**Figure 6:**
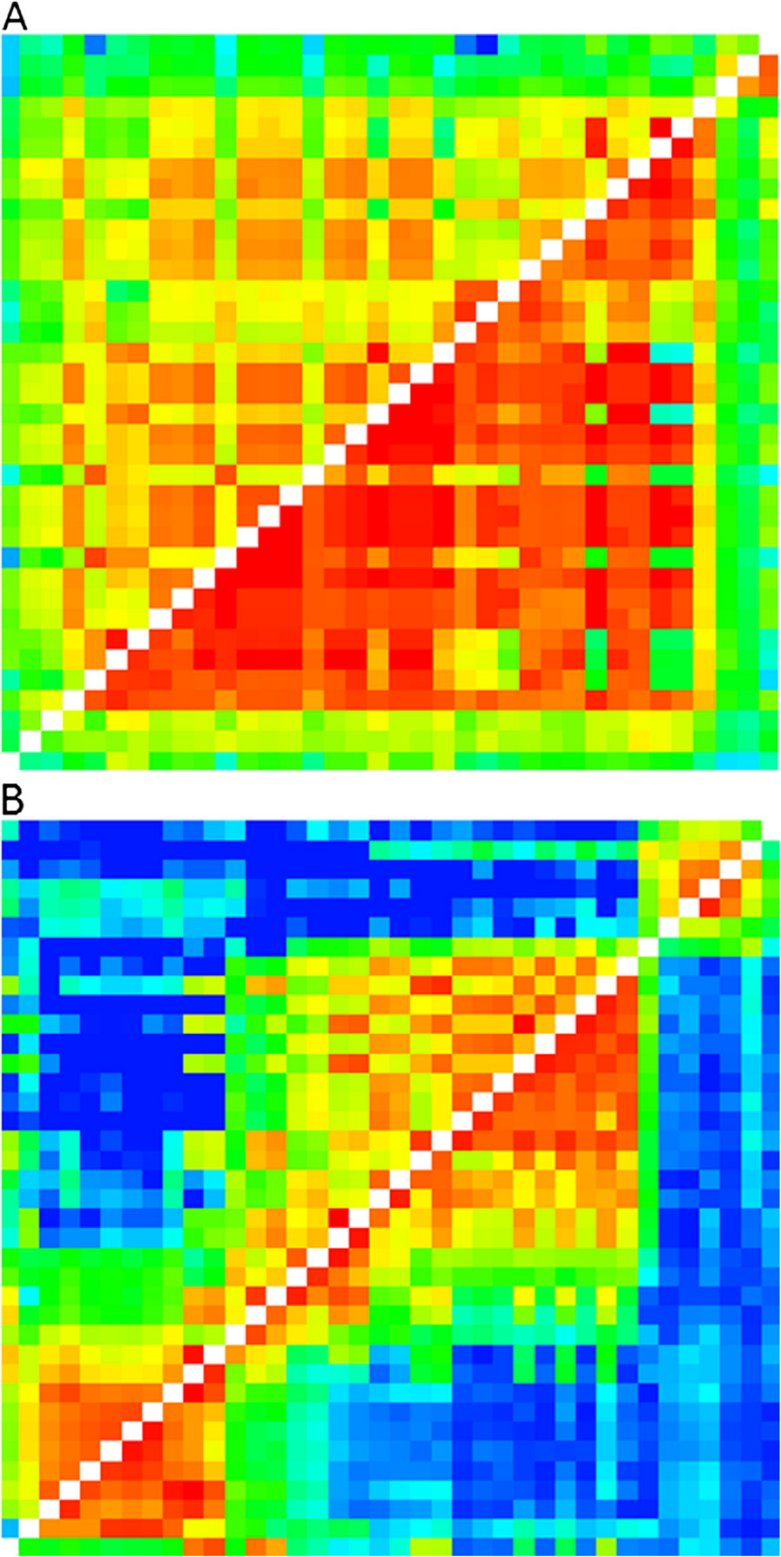
**GOslim annotations for assembled paired-end-RAD tags, annotated against the Swiss Prot database. A**. Distribution of GOslim annotations for biological process. The two most common processes are cellular transcription and transport. **B**. Distribution of GOslim annotations for cellular process. The most common terms are DNA binding and protein binding.

After removing duplicate sequences, including the second allele in heterozygous loci, 3,124 contigs remained. A BLASTX search of these contigs against the Swiss-Prot database identified 669 sequences that match a database sequence with an e-value of 10^-4^ or less. After assigning GOslim terms, the sequences span a range of biological processes and cellular functions (Figure 6A, Additional files 1, 2, 3, 4 and 5 ). Of the 669 annotations, 115 could not be assigned GOslim terms. While there were no specific patterns in the distribution of GOslim terms, the two most common processes are transport and transcription. Among the sequences with assigned GOslim terms the most common biological processes are cellular transcription and transport, followed by multicellular organismal development. In cellular function, DNA binding and protein binding were the most common terms with 18%, and 17% respectively (Figure 6B). Close inspection of the DNA binding category revealed numerous transposable elements. Additional transposable elements were found in the annotated sequences lacking GOslim terms. In total, out of 669 annotated sequences, 83 were transposable elements or related to transposition (12%). These were the most common annotations found in our data (the next most frequent is zinc finger proteins (17/669).

## Discussion

Here we present a first-pass meiotic map for sockeye salmon that contains 29 linkage groups, the number expected based upon the sockeye salmon karyotype (2n = 58) [42]. The final map contains 1,718 SNP and EST loci distributed over more than 4,000 cM.

Linkage maps have been created for a number of salmonids using a variety of genotyping strategies. Until now, these maps generally required iterative attempts, through time, where markers were added and linkage groups converged as new genotyping techniques materialized. In many cases these early efforts were based on microsatellite and amplified fragment length polymorphisms (AFLPs) and had modest initial map sizes. Currently the most complete linkage maps are available for Atlantic salmon (more than 5,000 SNPs, microsatellites, and AFLPs) [22] and rainbow trout (more than 4,000 SNPs, microsatellites, and AFLPs) [11,16]. The initial map in coho salmon *O. kisutch* contained 281 microsatellite and AFLP markers [43]. Mapping in brown trout *Salmo trutta* used 301 microsatellite and AFLP markers [44], and a map in arctic char *Salvelinus alpinus* contained 326 AFLP, microsatellite, and SNP markers [45]. The pink salmon *O. gorbuscha* map of 460 loci emerged from iterations that also included fragments originating from PCR priming on short interspersed repeats SINEs, [46] and randomly amplified polymorphic DNA RAPDs, [47,48]. Microsatellites have advantages over the other marker types due to their high rates of heterozygosity and large number of alleles which facilitate mapping. However all of these strategies are based on the accurate sizing of specific fragments and yield genotypes that are extremely difficult to compare among laboratories and through time.

Recently, massively parallel sequencing has allowed the rapid development of hundreds to thousands of novel SNPs in many species. As in our study, these studies map hundreds to thousands of novel markers. The rapid advance of high throughput sequencing promises to greatly speed the generation of novel genetic maps for previously unmapped or minimally mapped species [1,11,49,50]. The sequences from GBS are also easily converted to other methods of SNP genotyping, and genotypes obtained from SNPs from any method are generally comparable among laboratories and through time because they are based upon specific nucleotide differences rather than allele sizes.

Our map approaches the size of certain recent Atlantic salmon and rainbow trout genetic maps. The most recent Atlantic salmon map contains more than 5,000 SNPs [22]. The previous map for Atlantic salmon was also large, containing approximately 1,500 AFLP, microsatellite, and SNP markers, similar to our map size [51-54]. The most recent rainbow trout maps contain 1,124 markers based on AFLPs, microsatellites and SNPs [16] and 4,563 markers based upon RAD tags [11]. The AFLP and microsatellite-based maps represent years of discovery and mapping.

Salmonids have different rates of recombination between the sexes [7,43,44,54]. Rexroad et al. [55] reported an overall ratio of female to male recombination in rainbow trout of 1.68:1, with recombination rates on individual chromosomes ranging from 0.73:1 to 12.22:1. In Atlantic salmon the overall recombination ratio was 1.38:1, and the individual female to male ratios varied from 0.88:1 to 7.39:1 [22]. Direct comparison of our male and female maps is inappropriate because our maps contained both shared markers and markers unique to each gender. Given that marker order is generally conserved in the shared markers, we compare the distances between the most proximal and distal of the shared markers between the male and female linkage groups to compare rates of recombination. Overall recombination rates in males were lower than in females, which can be observed in Figure 5. The recombination ratio among shared markers on individual linkage groups ranged from 0.71:1 to 1.71:1 (Table 4). Our findings are consistent with those in both rainbow trout and Atlantic salmon where a number of the male linkage groups were larger than the female groups [16,22]. Current research in salmonids suggests that recombination in males is restricted to the telomeres [16,22], thus our values are likely underestimates of the true recombination differences between males and females (See also below).

There are regional differences in recombination between the females and males (Figure 4). In the female, markers recombine along the length of the linkage groups, while there are blocks of markers with little or no recombination in most of the male linkage groups. Large regions of non-recombinant markers have been observed in other salmonid species including Atlantic salmon and rainbow trout [11,16,22,53]. Two of these studies suggest that these blocks of non-recombinant markers may be located near the centromeres [11,53]; however, additional information is needed to confirm this assertion in sockeye salmon. While blocks of non-recombinant markers were present on most (23 out of 29 have regions of limited recombination) linkage groups in the male map, the blocks that we observed were smaller than those observed in Atlantic salmon or rainbow trout. Additionally, while overall recombination is reduced in the male as compared to the female, many of the markers shared between the genders are well spaced in both the male and the female (not contained within large, non-recombinant blocks). This may be an artifact of the OneMap analysis. OneMap uses maximum likelihood to assign phase to markers which are identical heterozygotes in the progeny and parents [56]. This allows the inclusion of these markers in the linkage map but it is unclear how this analysis may interact with the limited recombination in the male However, despite the spacing of these markers, overall the male map had more limited recombination than the female (see Figure 5). Inclusion of more families in future efforts would help to resolve marker spacing and order issues.

Our total map lengths are larger than previously observed in salmonids. The salmon genome size is estimated to be 2.4 to 3.0 × 10^9^ bp [57] which would correspond to a map length of approximately 3,000 cM (1 cM is generally estimated to be approximately one million bp). Several linkage groups such as LG6-F (Figure 4) contain large distances between either individual markers or small clusters of markers and the remaining linkage group. These large breaks could be the result of weak linkage relationships. Additionally, four linkage groups contained discrepancies in the order determined between male and female (Additional files 6 and 7). Varying the LOD score had no effect on the assignment of markers to their specified group. Other efforts to resolve the order including dropping and using the try.seq function on each marker to optimize position were unsuccessful. While the order could be manually changed, the size of the linkage group was often greatly increased and large breaks between markers were introduced. Increasing the number of individuals mapped may reduce the distance between these and other markers by strengthening linkage relationships and eliminating error, resulting in more consistent measurement of genetic distance, as well as helping to resolve marker order in the four linkage groups. Inflated map lengths and marker order problems may also be a result of missing data or genotyping errors [58]. Slate et al. [58] demonstrated that, when genotyping error is present, markers tend to be assigned to correct chromosomes but with inflated map distances and order errors. We minimized genotyping error in several ways: our RAD data coverage is higher than the recommended coverage values for accurate GBS [15,59], and markers with greater than 25% missing data, inheritance error, and significant segregation distortion were removed. However some errors may still remain. The effect of even a few errors is likely also compounded by our relatively small sample size (96). Increasing sample size to increase the number of recombination events would reduce the effect of genotyping error and give higher confidence in map lengths and order.

Our RAD tag loci, including both putative SNPs and monomorphic loci, were compared with those discovered for rainbow trout in Miller et al. [11]. Using strict comparison criteria, we discovered more than 16,000 matches between the two data sets, including 46 shared markers between the maps (Table 5, Additional files 1,2,3,4 and 5). These matches represent only 42 percent of the total number of the rainbow trout RAD loci. A previous study reported 95.7 percent identity between rainbow trout and sockeye salmon; however, this study focused on similarity among coding regions [20]. We compared anonymous regions of genomic DNA where rates of nucleotide divergence may be higher than those in coding regions. Due to the short length of the RAD tags, strict criteria for matches were imposed (full length, no more than 2 mismatches). This likely underestimates the number of true hits between the species. Salmonid species including rainbow trout, Atlantic salmon, arctic char and coho salmon have been shown to have large syntenic blocks among chromosome arms [43,44,51,60]. Our results are consistent with this finding. A recent study by Faber-Hammond et al. [61] demonstrated that the sockeye salmon neo-y chromosome corresponds to two rainbow trout chromosome arms from rainbow trout chromosome 8 and chromosome 2. Based on our comparison to the rainbow trout RAD map, we have corresponding matches on linkage groups LG9 and LG14 suggesting that one of these may contain the sockeye salmon neo-y chromosome. Further testing is needed to confirm this.

Longer contigs were assembled for more than 3,000 RAD tag alleles, and from these 669 (~22%) were annotated using a BLASTX search. We initially anticipated a much lower rate of annotation consistent with the percentage of protein coding sequence in salmonids (5% c.f. Cheng et al. 2005). Amores et al. [1] points out that *SbfI,* the enzyme used to create our RAD libraries, targets GC rich regions and thus cuts frequently in coding regions. Out of these 669 annotations, 83 (12%) were transposable elements or related to transposition. Transposable elements are common in large genomes [62] and it has been proposed that transposable elements may play a role in the speciation of salmonids [60,63]. The remaining annotations span a variety of biological processes and functions (Figure 6).

SNPs detected in RAD tags presented here should convert to high-throughput assays such as 5’-nuclease with high success. High-throughput genotyping of SNPs has emerged as an important tool for study of early life history, migration, and conservation of non-model organisms including sockeye salmon [64-66]. Many SNPs discovered in transcriptome sequence, an approach commonly used in the past, fail to amplify in high-throughput assays because of their proximity to intron boundaries [67]. We attempted assays for 10 of our RAD-based SNPs; nine successfully amplified and performed in three test populations (Additional files 1,2,3,4 and 5). Using SNPs derived from paired-end sequences enhances the opportunity to develop assays that require priming and probing templates that may approach 90 bp.

## Conclusions

Currently, genomic resources for sockeye salmon are scant. Here we present a first pass meiotic map generated from 1,772 RAD and EST-based SNPs. This map and set of SNPs will prove to be an enormous resource for both association mapping and landscape and conservation genomics e.g., [12,68]. Of course, adding RAD tags and EST-based SNPs for more individuals and more families will further refine the map and improve its value for comparative mapping.

## Methods

### Animals

Thirteen pairs of sockeye salmon from Lake Aleknagik, Alaska, were collected during August, 2009, and used to create 13 full-sib families. Axillary fin clips were taken from all adults and preserved in ethanol for DNA extraction. All embryos were incubated, first in Alaska, and later at the hatchery facility at the University of Washington. After hatch, juveniles were reared in recirculating aquaria for one month at which time 100 individuals from each family were sampled as whole fry and stored in RNA*later* (Ambion, Inc., Foster City, CA). Remaining individuals from each family were stored in ethanol. All animal care and use was carried out using methods approved by the International Animal Care and Use Committee (IACUC), under approved protocol 4229–01.

### DNA preparation

DNA extractions were carried out on all parents and offspring using DNeasy-96 kits (Qiagen, Valencia, CA) according to the manufacturers’ protocol. DNA was extracted from tail clips from 45–96 offspring from each family and fin-clips from all parents (Table 1). Concentration of the extracted DNA was assessed using the Quant-iT PicoGreen dsDNA Assay (Life Technologies, Carlsbad, California) on a Victor D plate reader (PerkinElmer, Waltham, Massachusetts).

### 5’-nuclease SNP Genotyping and Parentage test

All individuals were genotyped for 127 previously determined EST-based SNPs [64,69-71] following procedures for 5’ nuclease assays as described in Seeb et al. [72]. Genotype calling was carried out using the SDS 2.3 software (Life Technologies, Carlsbad California) or the BioMark 3.0.2 software (Fluidigm, South San Francisco, California). Monomorphic assays and assays which failed to amplify in the families were excluded from further analysis. The remaining 5’-nuclease genotypes were successfully filtered for Mendelian inheritance error and segregation distortion as described below, and markers uninformative for mapping were removed. Occasional family-to-family contamination had been previously observed in our fish incubation and rearing system.

### RAD library construction and sequencing

RAD library preparation was carried out on 96 offspring from one family and six parents (Table 2) using the methods similar to those previously described [39,40]. The parents were prepared for paired end sequencing to provide longer contigs for annotation and 5’nuclease assay design (see below); the offspring were prepared for single end sequencing. The methods are identical except for the size of the sheared library extracted from the gel, 400–800 bp for single end sequencing and 150–400 bp for paired end sequencing. The restriction enzyme *SbfI* was used to digest the DNA, and *SbfI* specific Illumina linkers each containing a unique barcode were ligated to each digested DNA sample as described in Miller et al. [11]. Individual samples were pooled into libraries containing up to 16 individuals. Each library assessed for quality and concentration using a Bioanalyzer DNA 1000 kit (Agilent Technologies, Santa Clara, California), and final concentration was determined by the Bioanalyzer software (Agilent Technologies, Santa Clara, California).

Ten nanomoles of each library in EB (Qiagen, Valencia, California) and 0.1% Tween 20 were sequenced at the University of Oregon High Throughput Sequencing Facility. The parents were sequenced using paired-end technology at 2 × 80 bp on the Illumina Genome Analyzer II (GAII). All offspring were first single end sequenced at 80 bp on the GAII. Another round of sequencing was required to achieve satisfactory depth of coverage; the second round was conducted on the Illumina HiSeq2000 (Table 3), which produces 100 bp reads.

### SNP discovery

All sequence analysis for parents and offspring was carried out using Perl scripts (Perl scripts used are available from Miller et al. [11] ) and the publically available software programs Bowtie version 0.12.7 [73] and Novoalign version 2.07 ( http://www.novocraft.com). First the sequences were filtered for quality. The last ten base pairs from each sequence were removed from GAII sequences (paired end and single end); subsequently any reads with less than 80% chance of being error free were discarded from further analysis. Sequences obtained from the HiSeq2000 were trimmed to 70 bp by removing the last 30 bp (to match the GAII sequences), and any reads with less than 80% chance of being error free were discarded. The 80% threshold was determined using the Phred quality score of each read. The quality value at each nucleotide was converted to a probability score and summed across the read length and if the sum fell below 80% the read was discarded. All quality filtered data was de-multiplexed into separate sequence files for each individual using the barcodes contained in the Illumina linker ligated during library preparation (see above) [11]. These procedures were applied to all GAII single end, GAII paired end, and HiSeq2000 data. The data for each offspring from the GAII and the HiSeq2000 was combined for all further analysis. SNP discovery was then carried out using only parental sequence data.

Using Perl scripts, the set of unique RAD tag sequences for each parent was identified, and the count of occurrences for each unique sequence within each parent was obtained. After labeling each sequence with the parent of origin, the set of sequences from both parents were combined for alignment in Novoalign. The combined set of RAD tag sequences were self-aligned in Novoalign. The software was set to carry out an exhaustive search, returning up to 40 alignments per unique sequence. We set a maximum alignment score of 125. The results of this alignment were filtered to maximize true (non-paralogous) loci shared between parents. These loci included monomorphic and SNP containing loci, as well as some paralogs. The loci were filtered using methods adapted from Miller et al. [11]. In order to filter the sequences, the script compares each alignment score to a threshold value, compares the number of reads at each alignment, and finally compares the number of alignments within and between each parent. The Perl script to identify loci takes into account alignment score, coverage, and the number of identified alignments both between and within parents. For this study a minimum alignment score of 30, with no more than one alternative allele in either parent was used. The results of this search were filtered to include only the sequences containing putative SNPs, and these were output in a FASTA file for use as a reference sequence for Bowtie alignment.

### SNP genotyping in offspring

The sequences containing putative SNPs in the parents were used as a reference set in a Bowtie alignment, formatted as “allele 1” and “allele 2” for each putative SNP. Quality filtered sequences from all offspring and the two parents were aligned to this reference, with Bowtie set to only align reads which matched the reference perfectly. The output of this alignment was filtered using custom Perl scripts, counting the number of matches to each allele for each individual. These allele counts were exported for conversion into genotype calls in Excel.

SNP genotype calls from the allele counts were made using methods similar to those in Nielsen et al. [59]. A threshold of 10 reads from both alleles (sum of both alleles) was required for genotyping; any locus with less than ten reads was called as missing. This threshold was determined experimentally by testing various threshold values on the data set and examining the resulting calls for inheritance error, segregation distortion, and the number of missing markers, and subsequently comparing these values between thresholds. During SNP discovery each allele was designated as allele 1 or allele 2. For all loci, allele 1 was designated as the reference allele, thus in some cases the alternative allele may have higher read counts than the reference allele. For each locus, a heterozygous genotype was called if the read count for the non-reference allele was between 28%-80% of the total count for both alleles. Otherwise a homozygous genotype was called. Certain combinations of allele counts may still result in a miscalled allele, however, such a miscall in the parents would result in a mismatch in alleles between parents and offspring. All data was screened for such inheritance error (see below) and markers with an excess of these errors were removed from the analysis.

Next, parent and offspring genotype calls were compared to check for inheritance error, where the genotypes of the progeny do not match the expected genotypes based on the parental genotypes. If found, these genotype calls were converted to missing data. The informative genotype calls from existing sockeye 5’-nuclease assays were added to the data set. The data was checked for segregation distortion from 1:1 or 1:2:1 ratio using a χ^2^test. Markers exhibiting significant levels of segregation distortion were removed from the analysis. Finally, markers for which one parent or more than 25% of offspring genotype calls were missing were removed, and the genotypes were converted into OneMap format [56,74].

### Linkage mapping

Linkage analysis was carried out using the R package OneMap version2.0-1 [74]. OneMap uses a maximum likelihood analysis to assign phase to identical heterozygous progeny genotypes, allowing the inclusion of these markers in the linkage map [56]. Male and female linkage maps were generated separately. All linkage map ordering was carried out using the Kosambi map function [8,75,76]. Data was imported in outcross format. Next, the recombination fraction between all pairs of markers was calculated using the rf.2pts algorithm. Linkage groups were formed using the group command in two stages. First, linkage groups were formed with LOD = 12 and a maximum recombination fraction of 0.01. These groups were ordered using either the compare function or the order.seq function depending on the number of markers. Groups with up to seven markers were ordered with the compare function, groups containing more than seven were ordered with order.seq. This identified markers which overlap in position. One marker from each position with the fewest missing genotypes was selected to use in the next step of map construction. Next, linkage groups were formed with LOD = 6 and the default recombination fraction. Order.seq was used to place markers in their initial order. After initial ordering, ripple.seq was used to test the marker order, with a sliding window of four markers, and the order was changed when necessary. The marker orders were also inspected visually using rf.graph.table, which plots a heat map of the LOD score and recombination frequency, for ordering errors too far apart to detect with ripple.seq and made manual changes when necessary. Finally, the markers removed in the LOD = 12 analysis were individually added back to each linkage group using the try.seq and ripple.seq functions to finalize marker order. The male and female linkage maps were compared for marker order among the shared markers. Where discrepancies were found the individual marker was removed using drop.seq, and then added back to the map using try.seq to examine optimal marker position. In cases where no consensus order could be established this is noted in the text.

Linkage relationships were obtained among the segregating 5’-nuclease loci in each of the 12 families and in both the RAD tag data and the 5’-nuclease assays in the sequences family. The individual male and female maps from each family were combined where possible using MERGE, part of the LINKMFeX package [77].

### Comparison to rainbow trout RAD tags

All putative loci discovered in the HX13-WL parents were compared to the rainbow trout RAD tags in Miller et al. [11] using BLASTN (BLAST version 2.2.25). For unmapped loci, only full length (59 bp) hits with no more than one mismatch were considered. For loci used for either map, the stringency was stepped down and the map locations were compared. If hits occurred in one to two linkage groups, consistent with syntenic matches, they were kept. In this way, hits longer than 56 bp and containing no gaps and no more than four mismatches were considered matches. Microsoft Excel was used to identify shared tags that were present in both rainbow trout and sockeye salmon linkage maps. Tag positions were compared between the rainbow trout and sockeye linkage maps and overlapping map hits were counted.

### Paired End assembly

Paired-end sequence data from the parents was assembled to generate longer sequences for use in annotation of RAD tags and as a resource for 5’ nuclease assay design. Paired-end assembly was carried out using custom Perl scripts and the publically available program Velvet version 1.1.06 [78]. The Perl script matches each sequence in the FASTA file generated during SNP discovery, containing the 6,860 SNP alleles, against the forward end (paired-end 1) paired-end file and pulls both matching sequences and their pairs and writes them into a temporary data file. Both alleles of each SNP discovered were used as the template for the paired-end sequence assembly. The complete set of paired, filtered reads from all six individuals was compared to the SNP-containing FASTA file. The temporary file is then run through Velvet, and the resulting output is added to the results file. This procedure is repeated iteratively until all the sequences have been compared. Velvet is set with a k-mer value of 21 and a minimum contig length of 150 bp. All other Velvet settings are set to default values. For any contig not meeting the 150 bp minimum, a placeholder value is inserted to indicate the missing sequence. After assembly, duplicate contigs were removed.

Contigs from the paired-end assembly were subjected to a BLAST search against the Swiss-Prot database using BLASTX. An e-value of less than 10^-4^ was required to accept the annotation. Microsoft Access and the complete set of GO terms for the Swiss-Prot database (NCBI version 2011_11) were used to associate sequence annotation with their GOSlim term. Terms were assigned for both biological process and cellular function.

The 5’-nuclease assays were designed as described previously ( [67,69,70]). Five assays were designed on assembled paired end sequence, and five assays were designed from RAD tags alone.

## Competing interests

The authors declare that they have no competing interests.

## Authors’ contributions

JES and MVE designed the study. JES conducted matings and incubated the embryos; MVE reared the fish and collected the genotypes. MRM designed the genotyping pipeline and contributed to the genotyping analysis. MVE designed the linkage analysis pipeline and constructed the maps. The paper was written by MVE and JES. All authors read and approved the final manuscript.

## Supplementary Material

Additional file 1 **Supplement 1a.** Female and male meiotic maps for sockeye salmon. Click here for file

Additional file 2 **Supplement 1b.** RAD sequences and annotation list from paired-end assembly. Click here for file

Additional file 3 **Supplement 1c.** 5’-nuclease assay information designed from RAD tags. Click here for file

Additional file 4 **Supplement 1d.** 5’-nuclease assay information for EST based SNPs used for mapping. Click here for file

Additional file 5 **Supplement 1e.** List of syntenic RAD tags between rainbow trout and sockeye salmon. Click here for file

Additional file 6 **Supplemental_File_2a.** Graphical figures of sockeye salmon meiotic maps. Click here for file

Additional file 7 **Supplemental_File_2b.** Graphical figures of sockeye salmon meiotic maps. Click here for file
