# Supplementary figures and images for: Meiotic maps of sockeye salmon derived from massively parallel DNA sequencing
Source: BMC Genomics. 2012 Oct 3;13:521. doi: 10.1186/1471-2164-13-521 (PMC3563581; doi:10.1186/1471-2164-13-521)

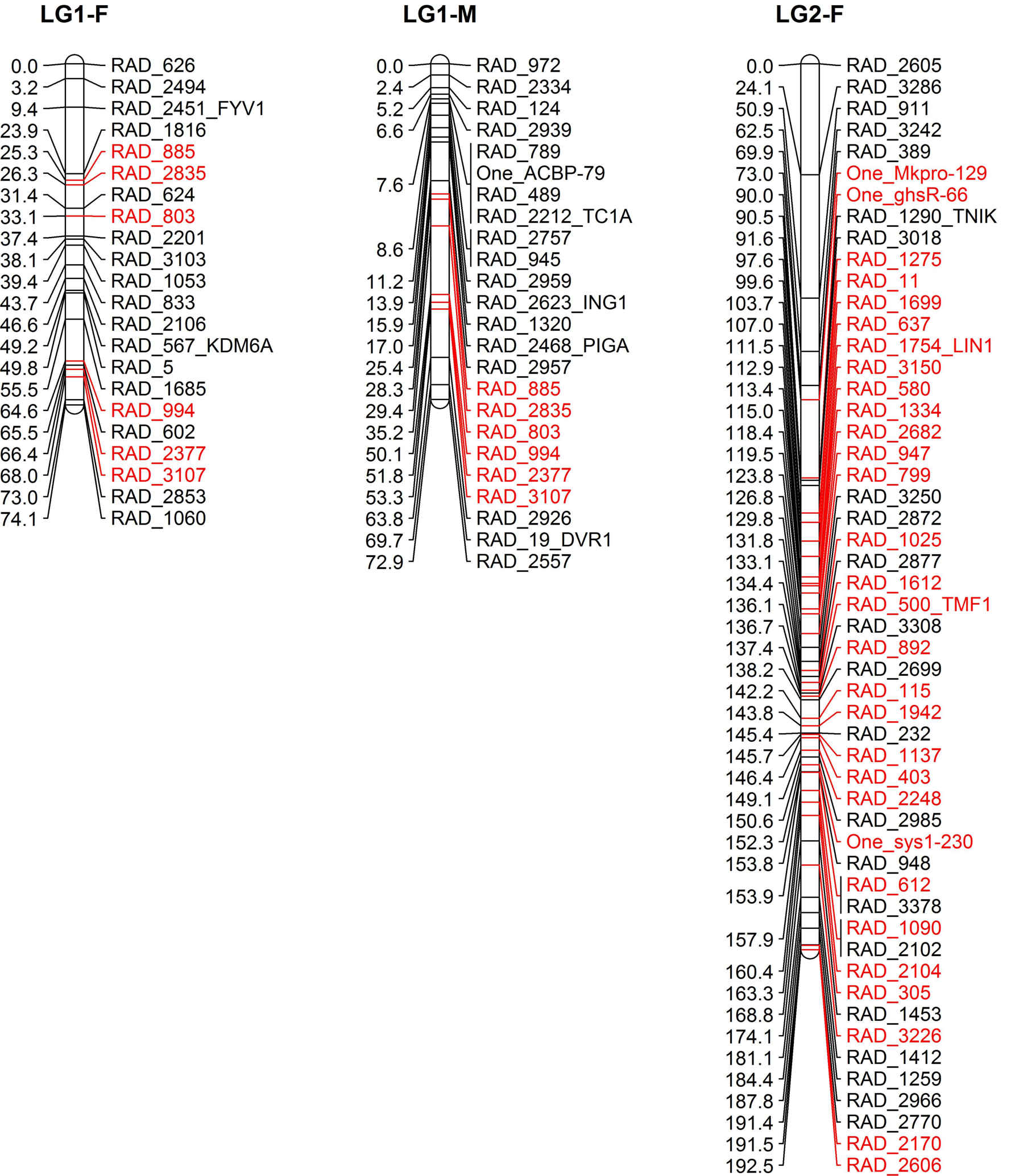

LG2-M

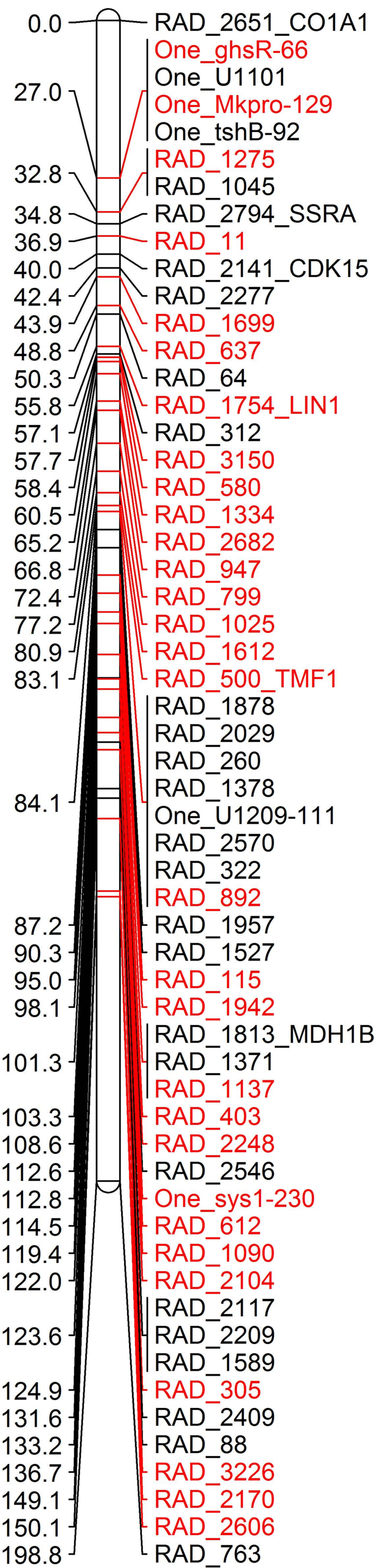

LG3-F

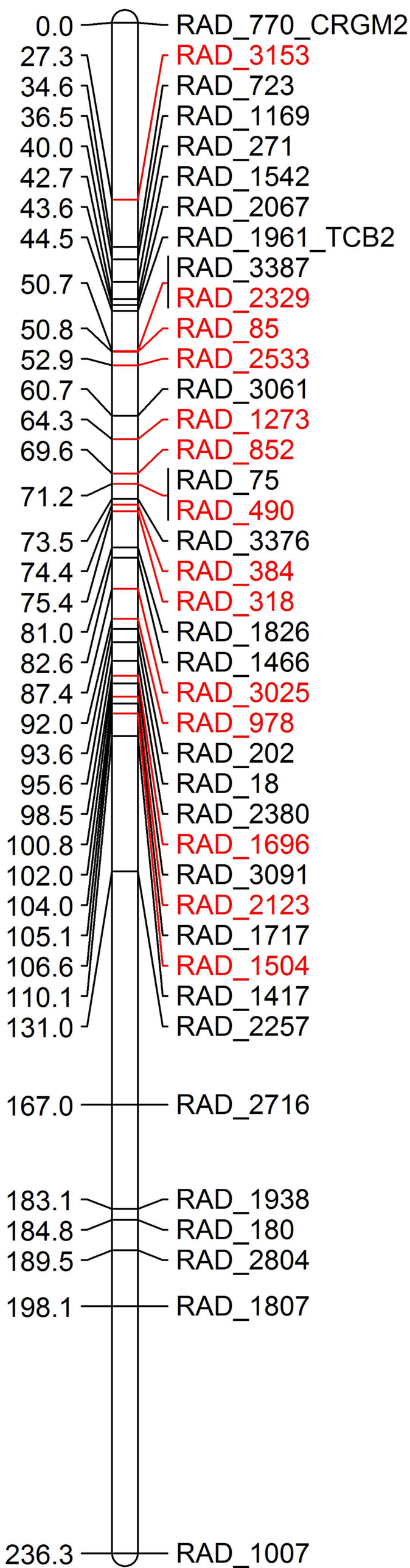

LG3-M

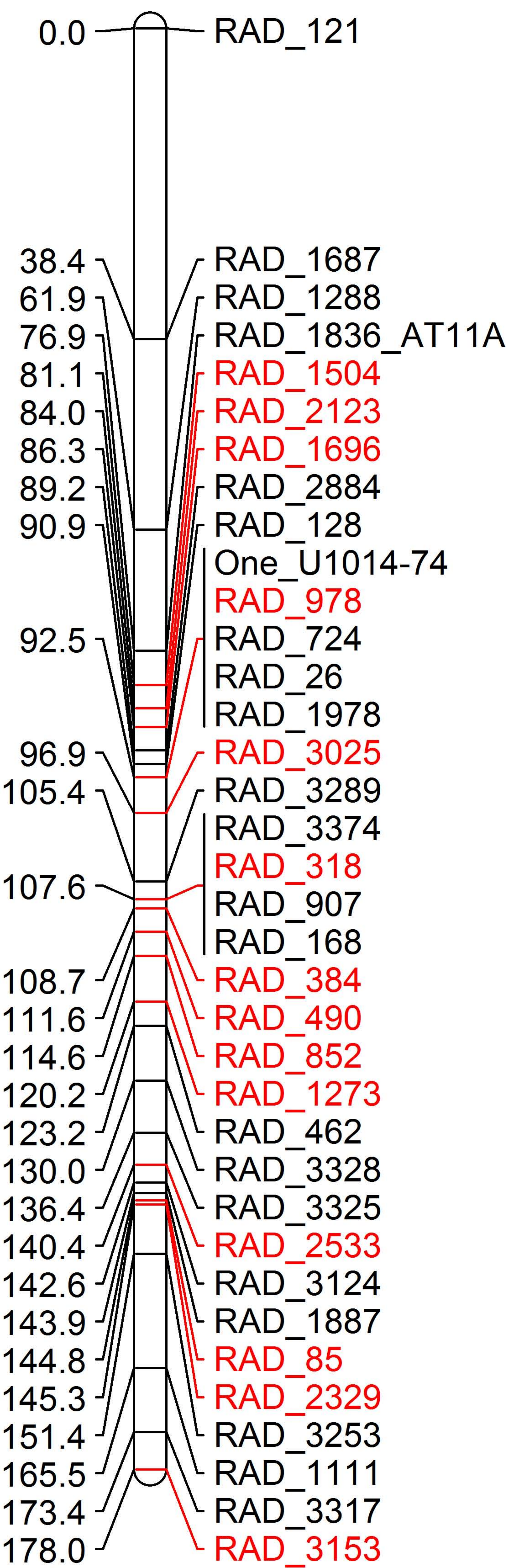

LG4-F

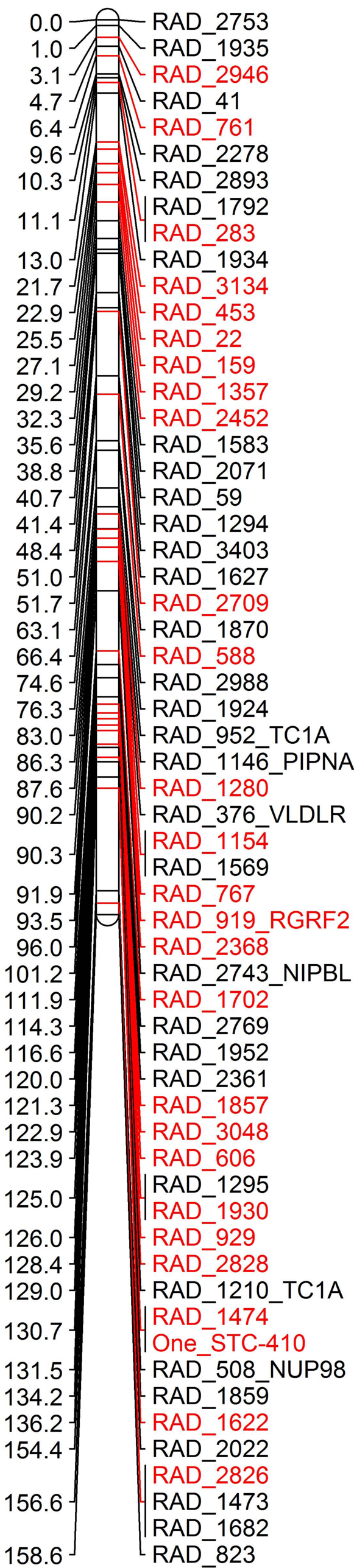

LG4-M

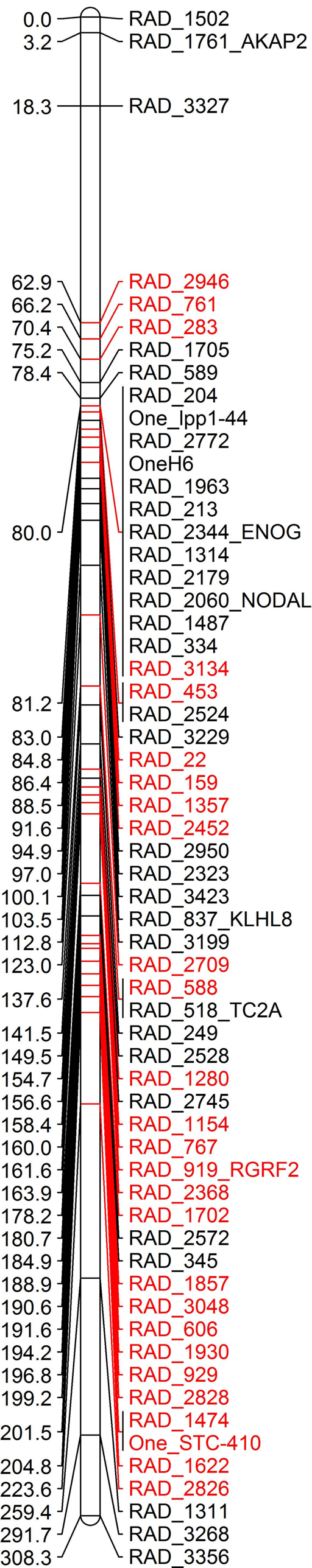

LG5-F

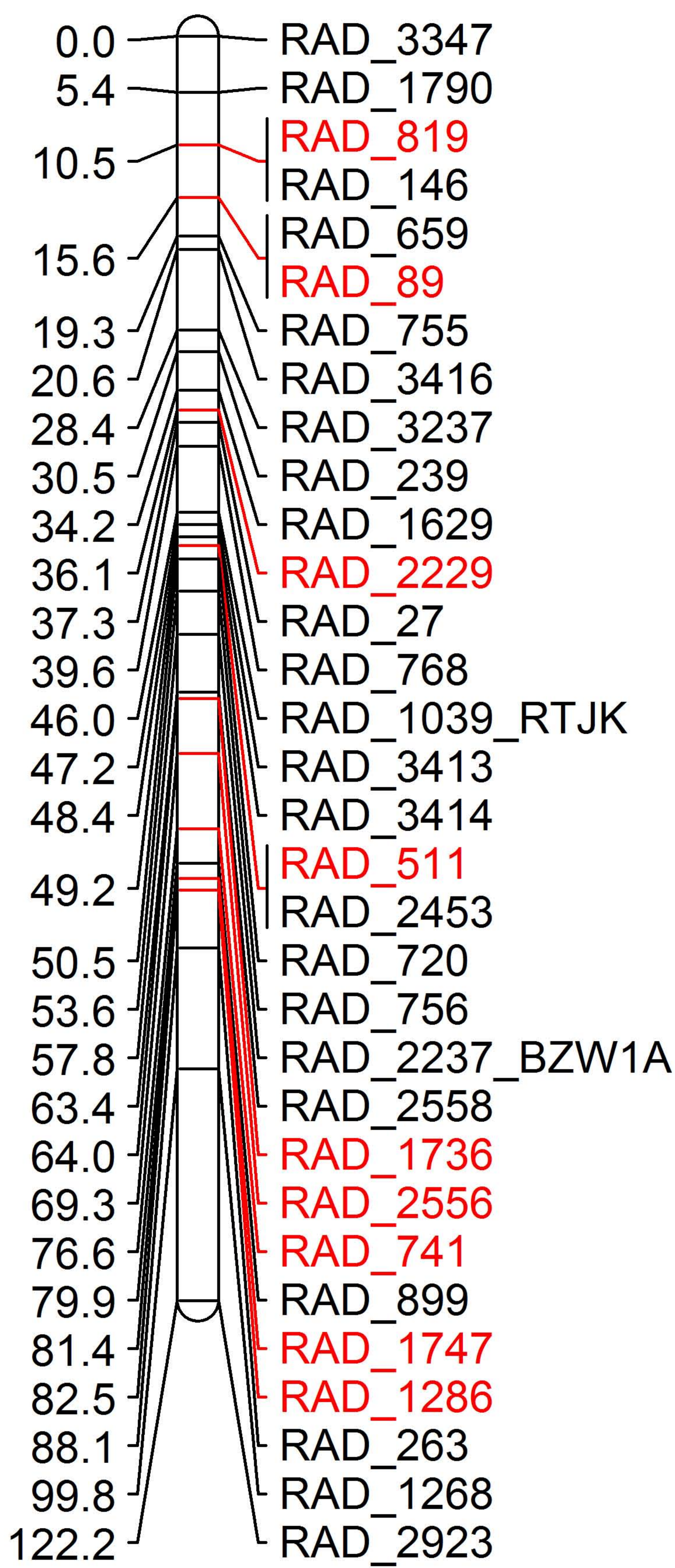

LG5-M

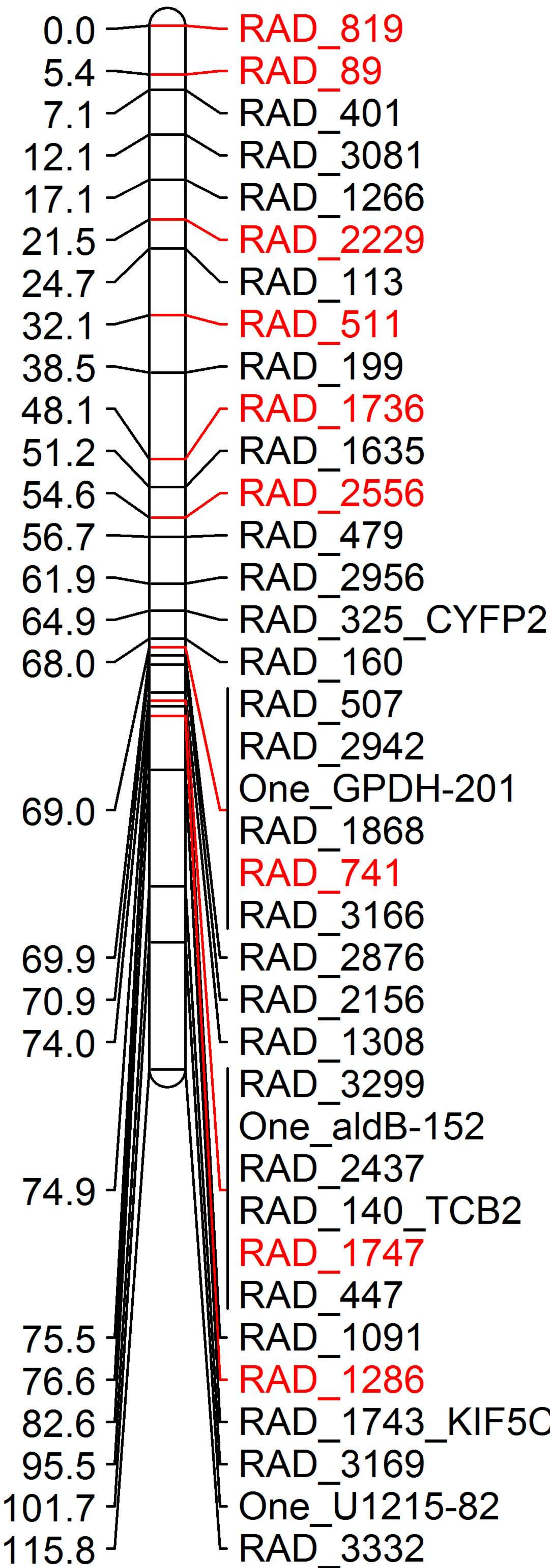

LG6-F

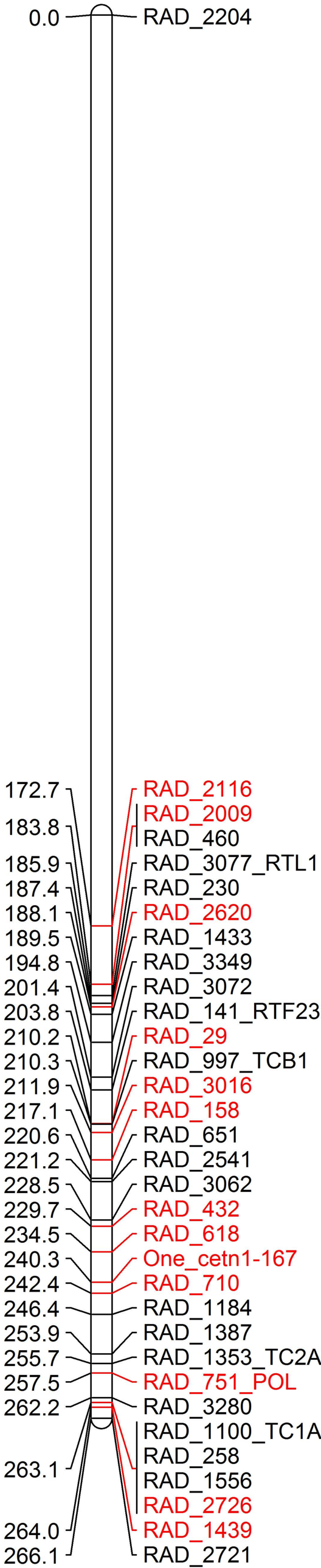

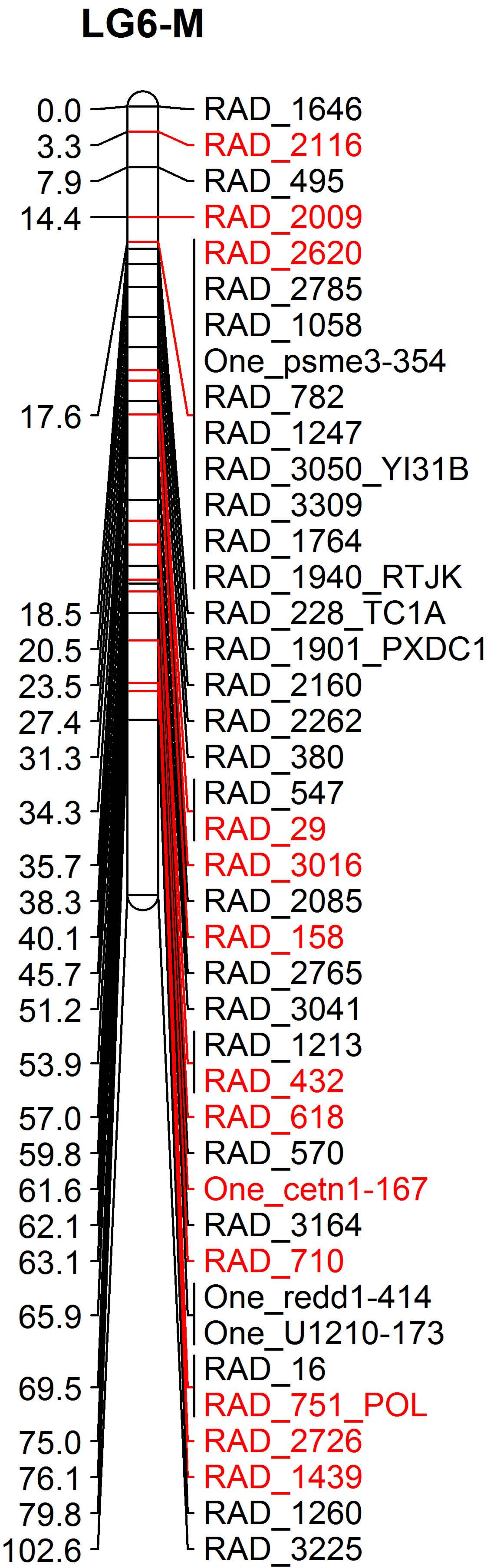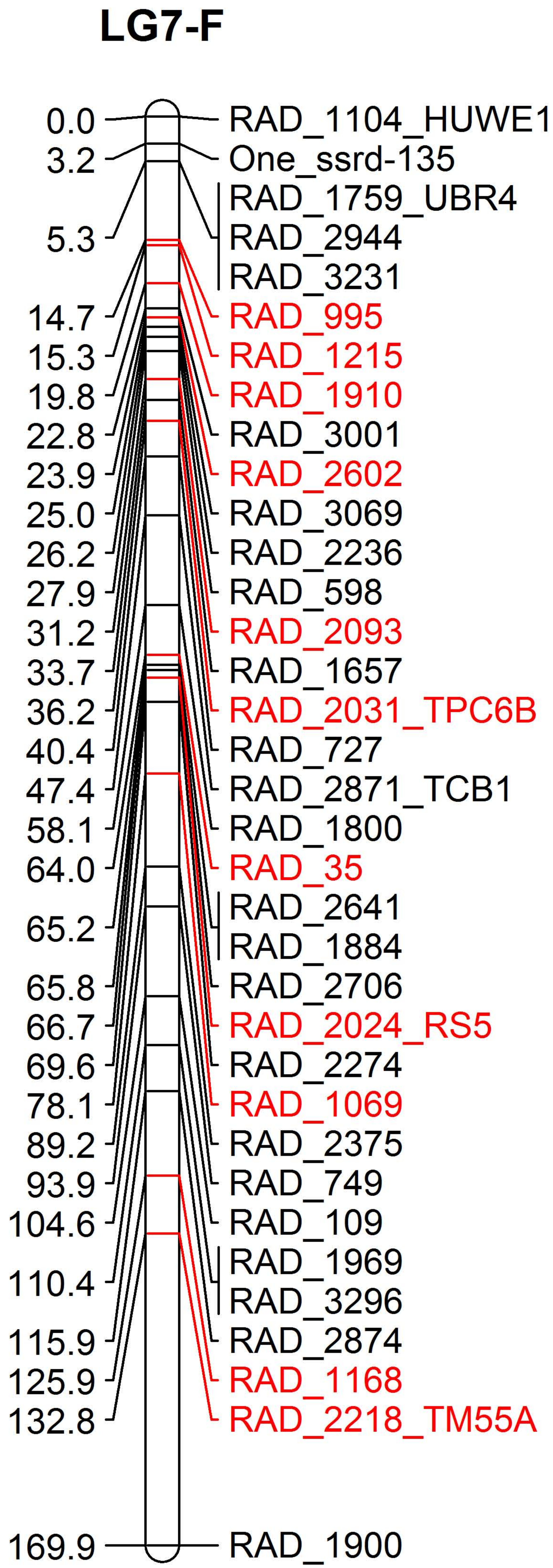

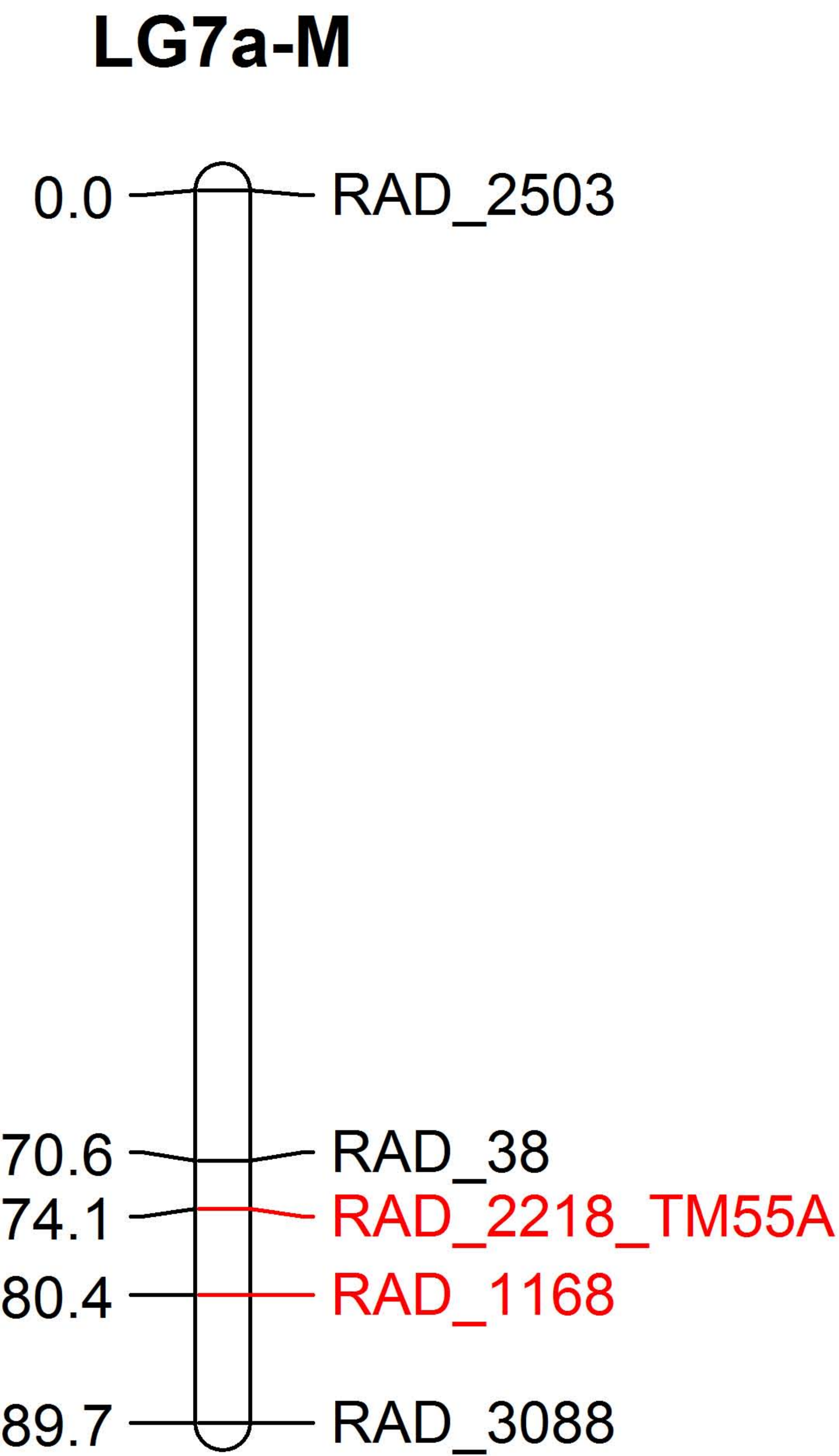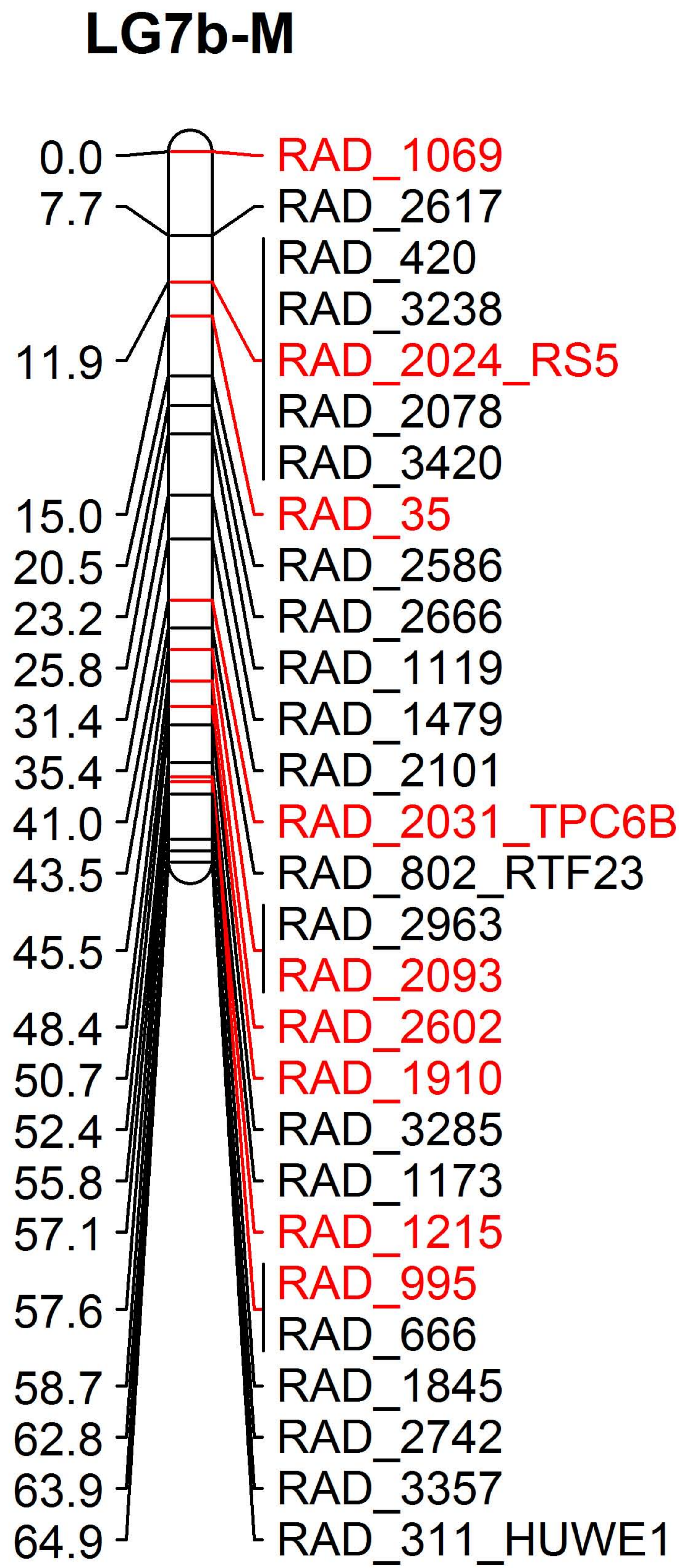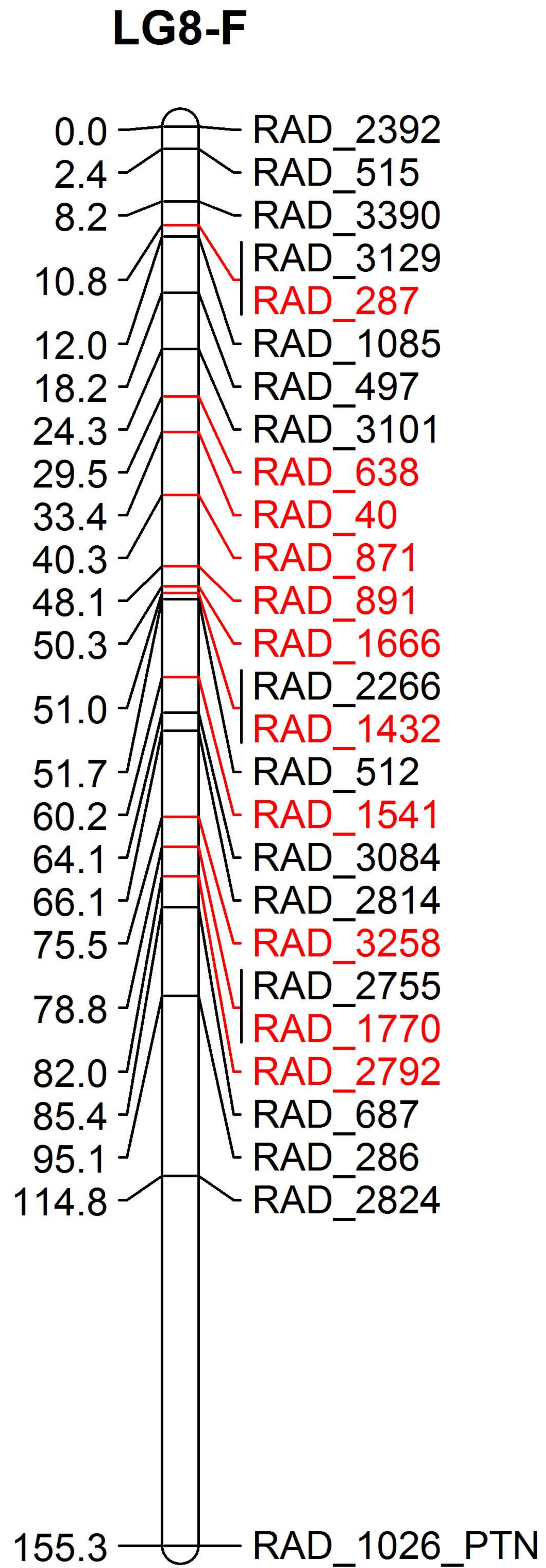

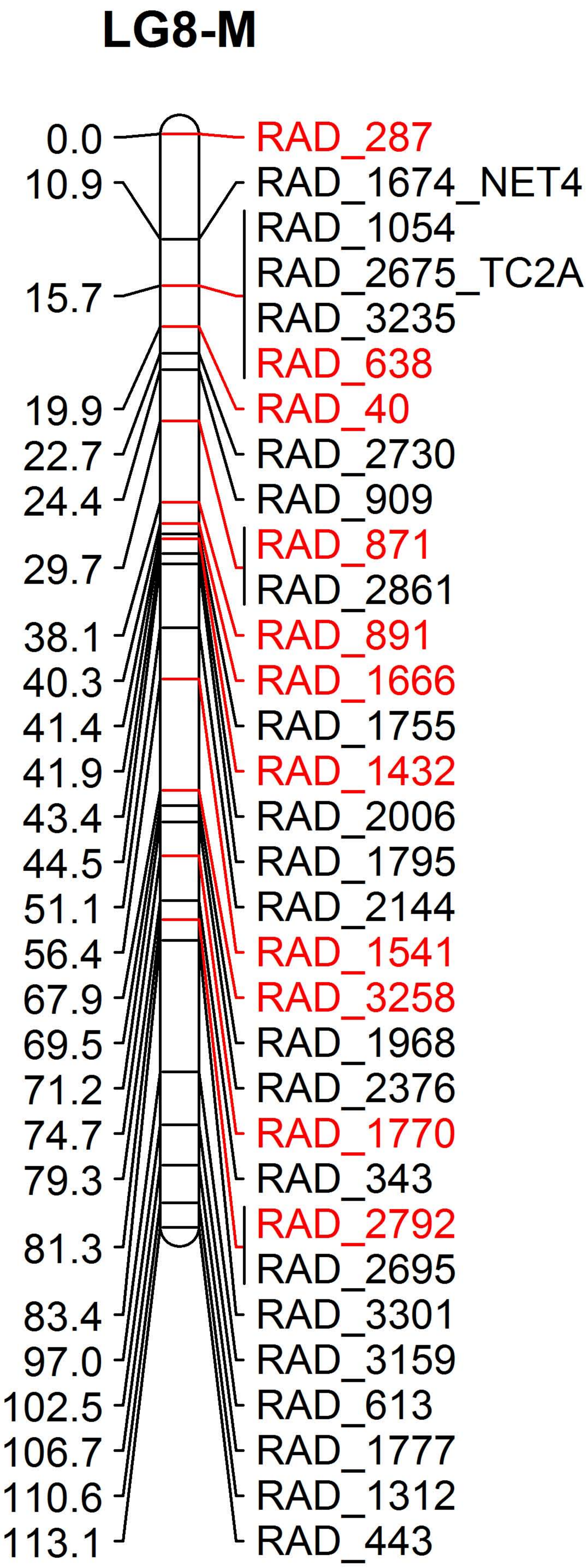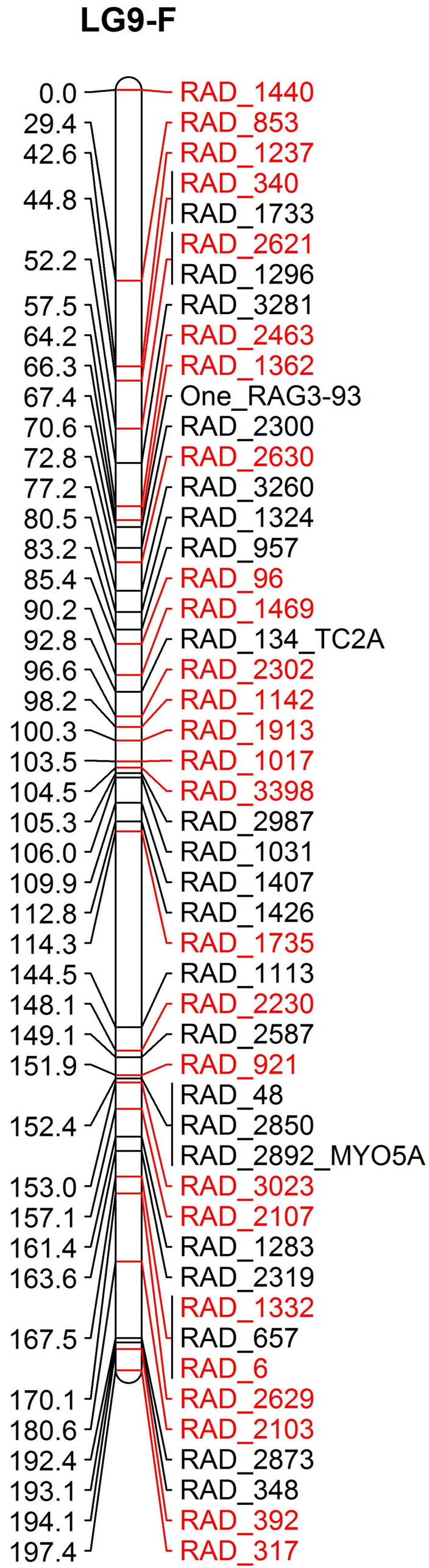

LG9-M

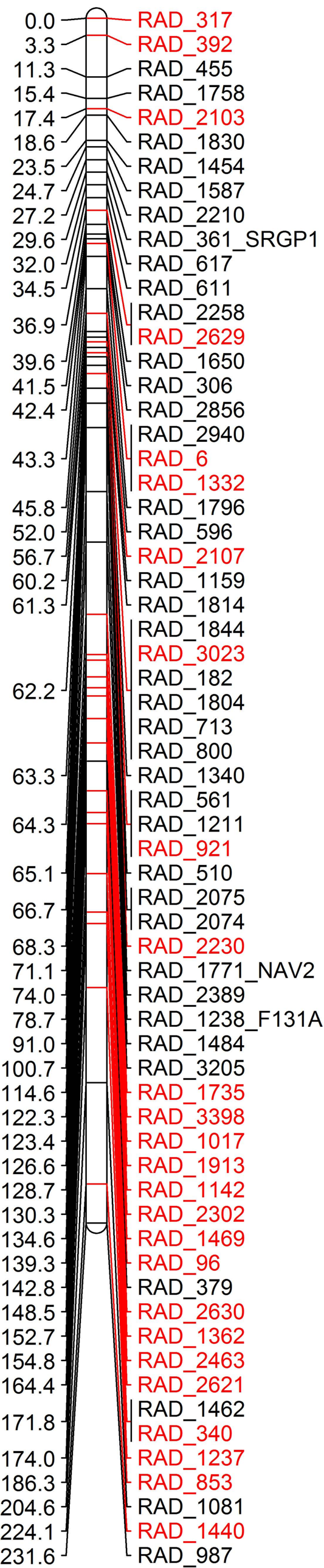

LG10-F

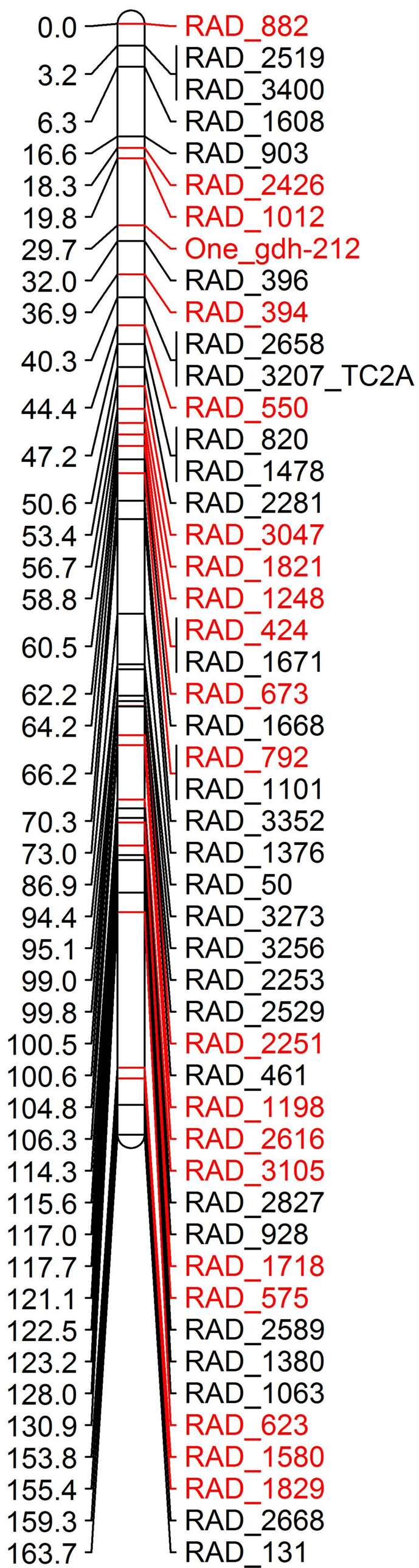

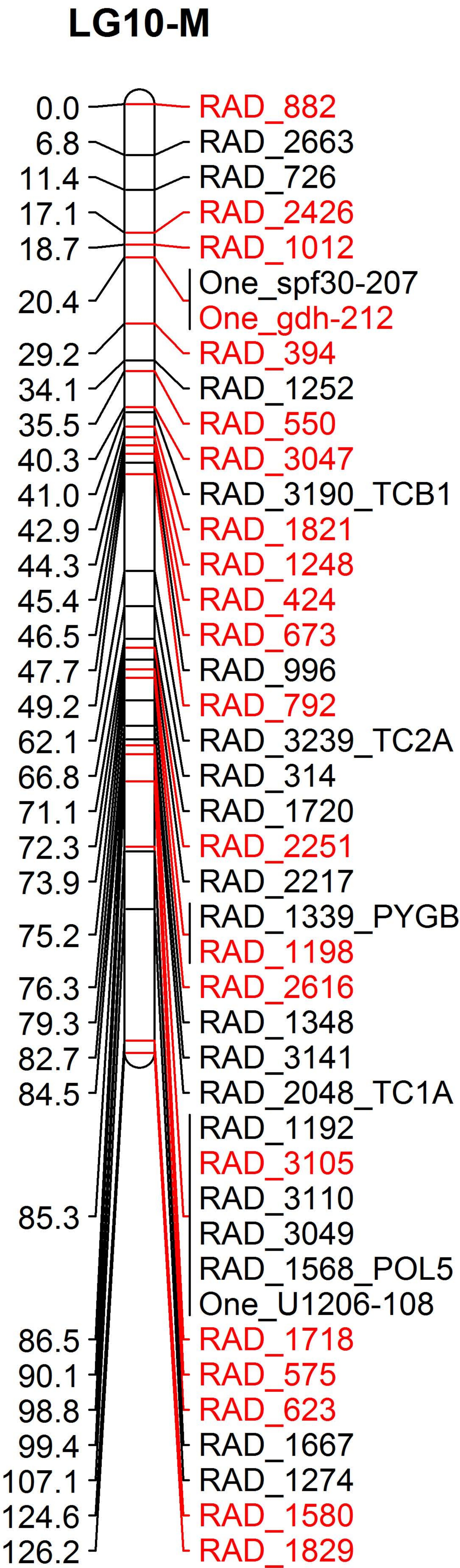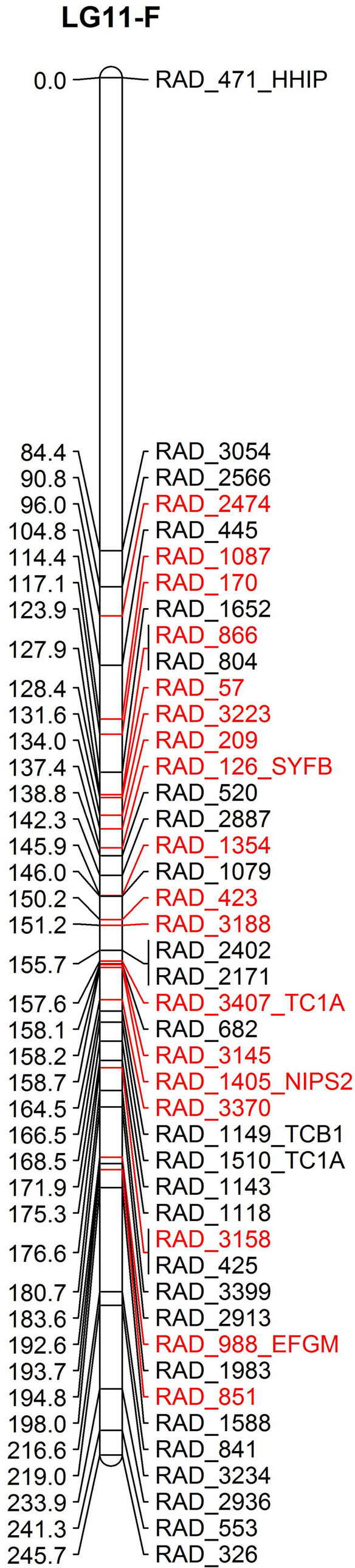

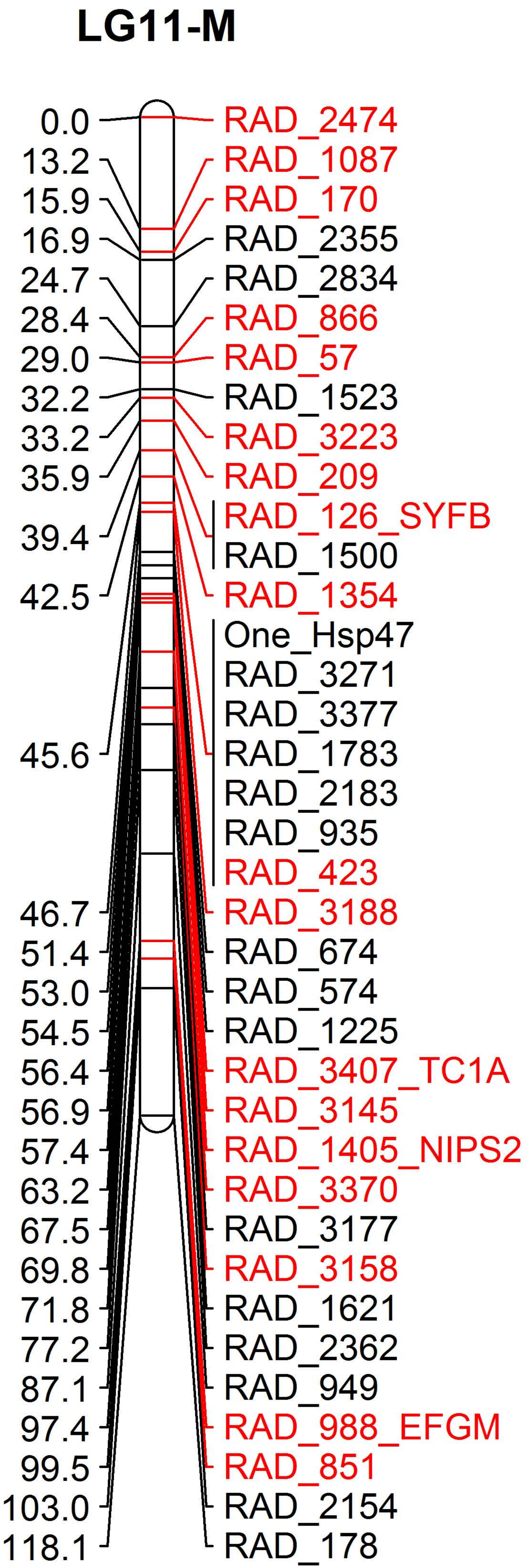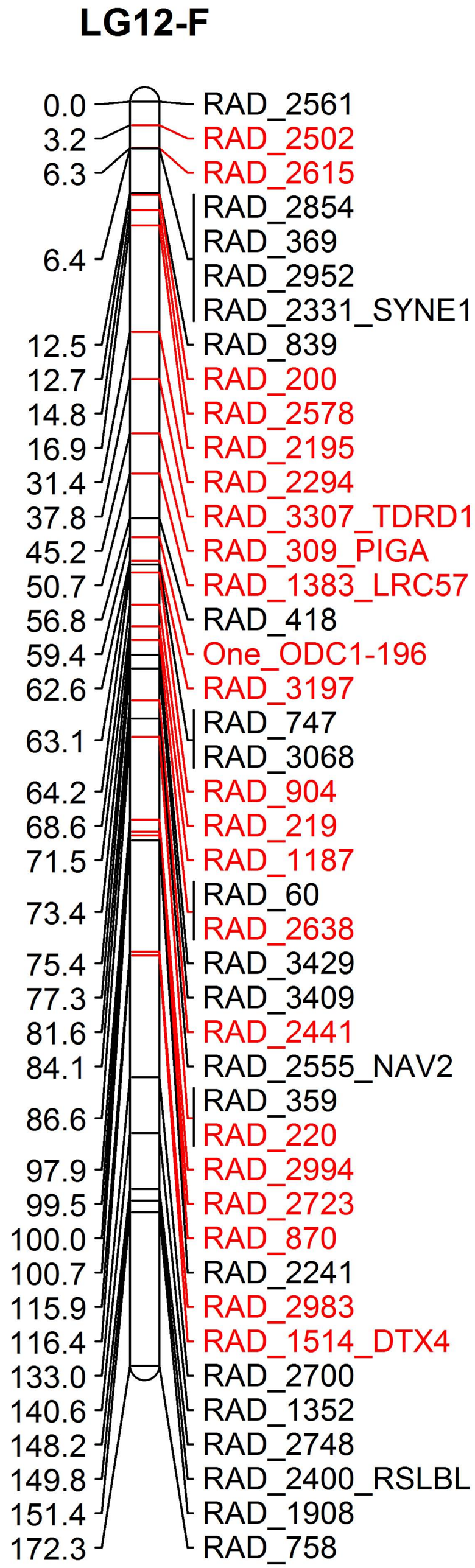

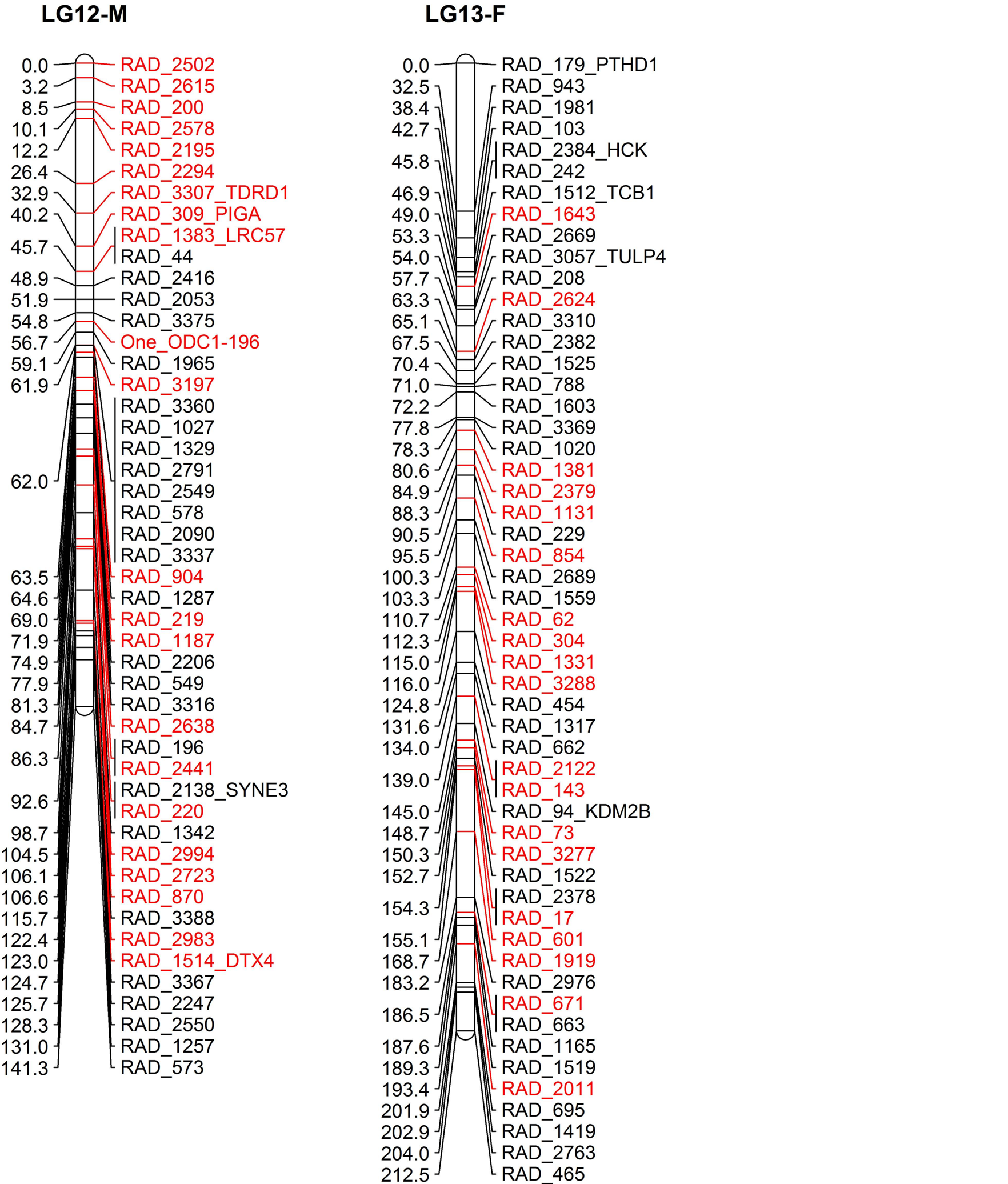

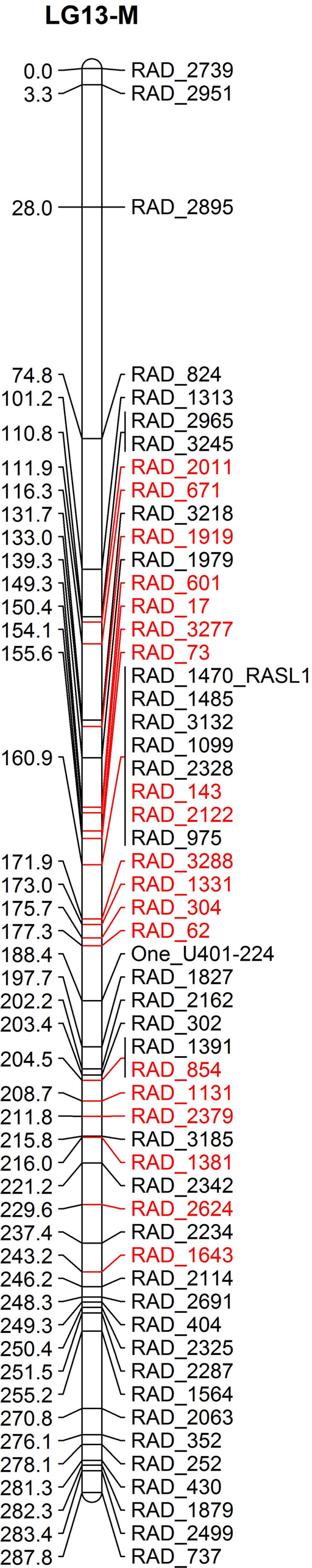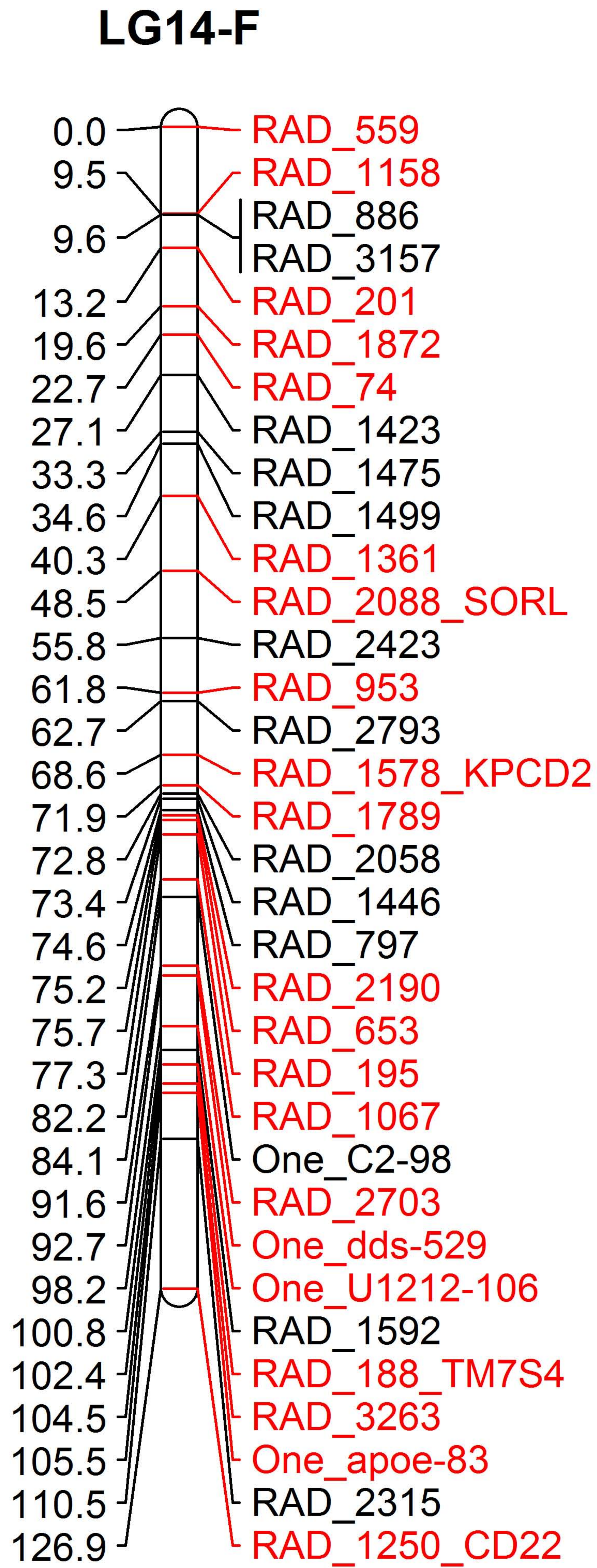

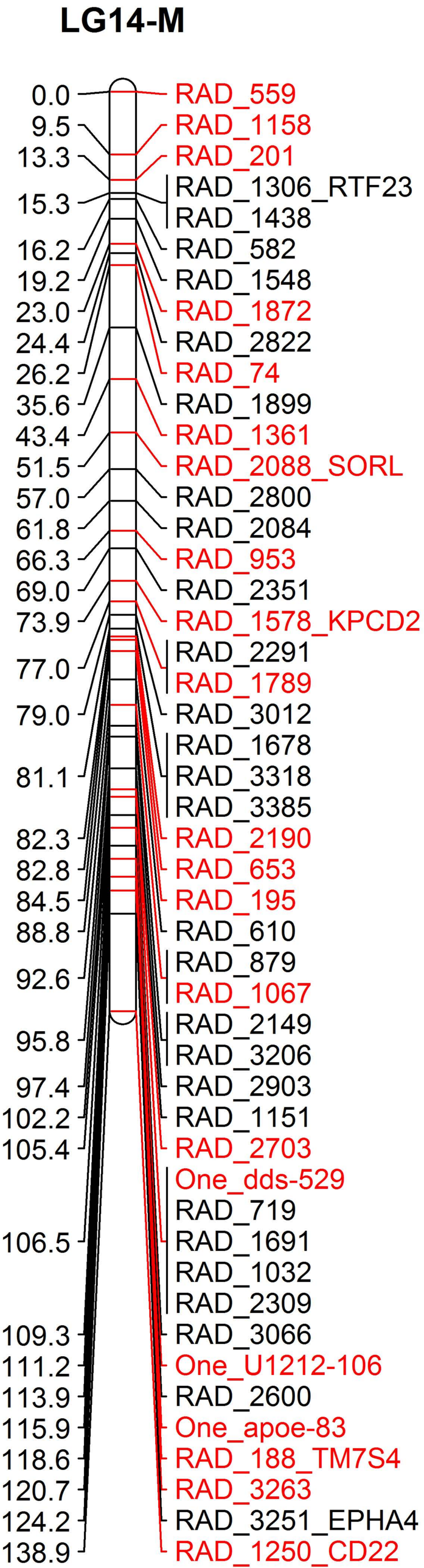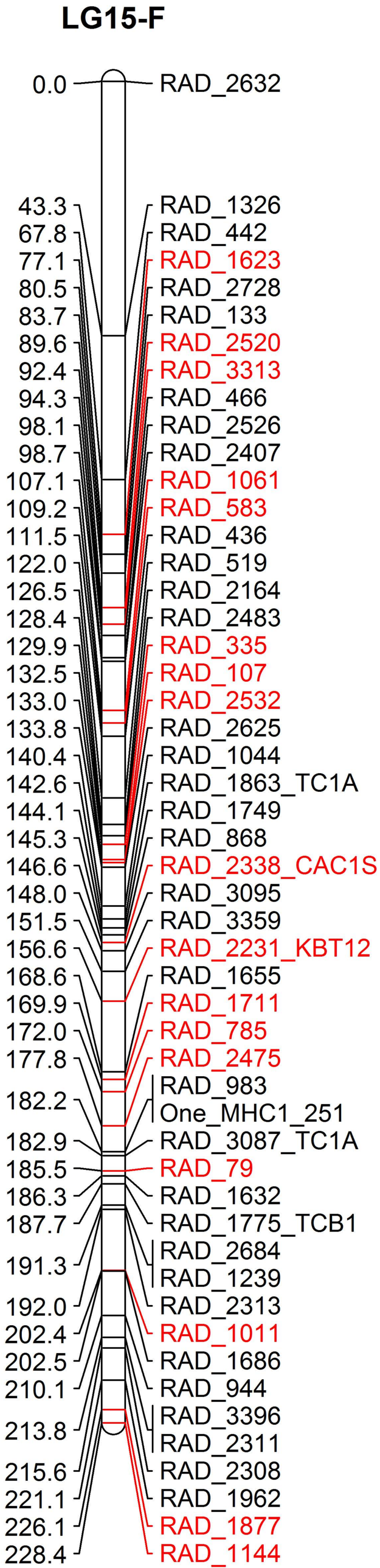

Supplement: Additional file 6 — Supplemental_File_2a. Graphical figures of sockeye salmon meiotic maps. [file 1471-2164-13-521-S6.pdf]

LG15-M

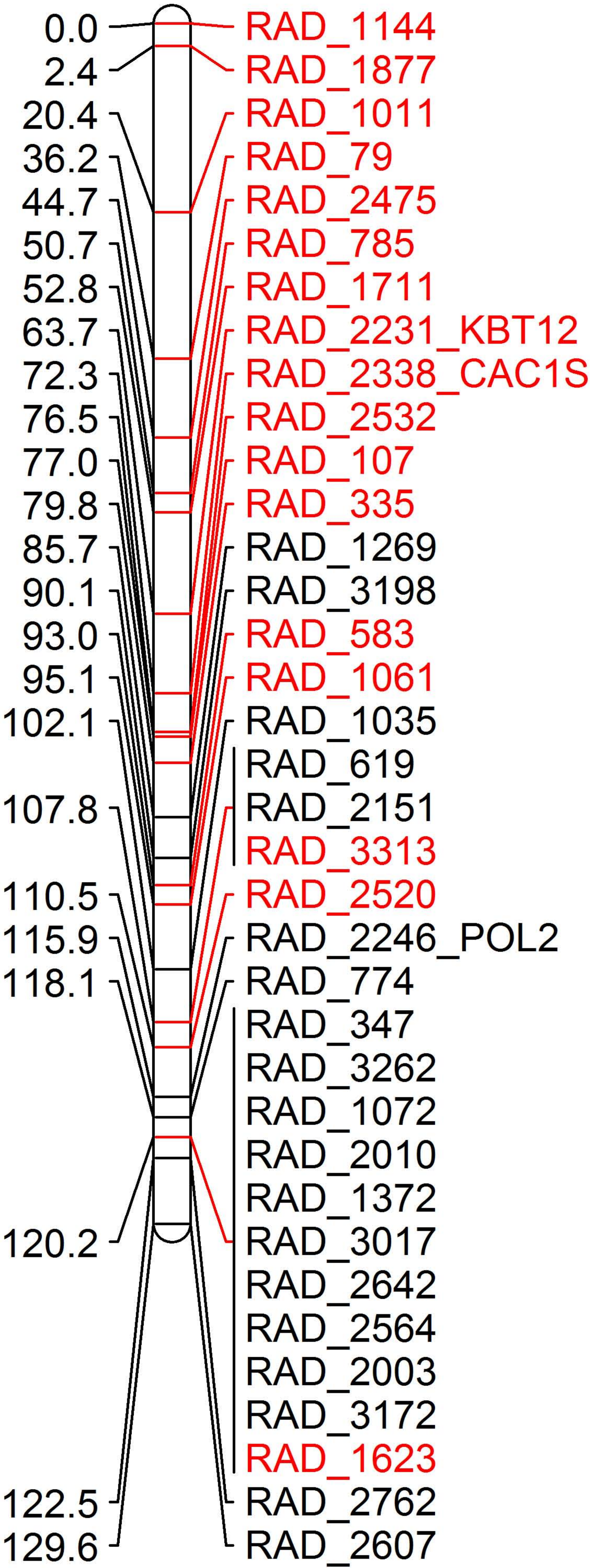

LG16-F

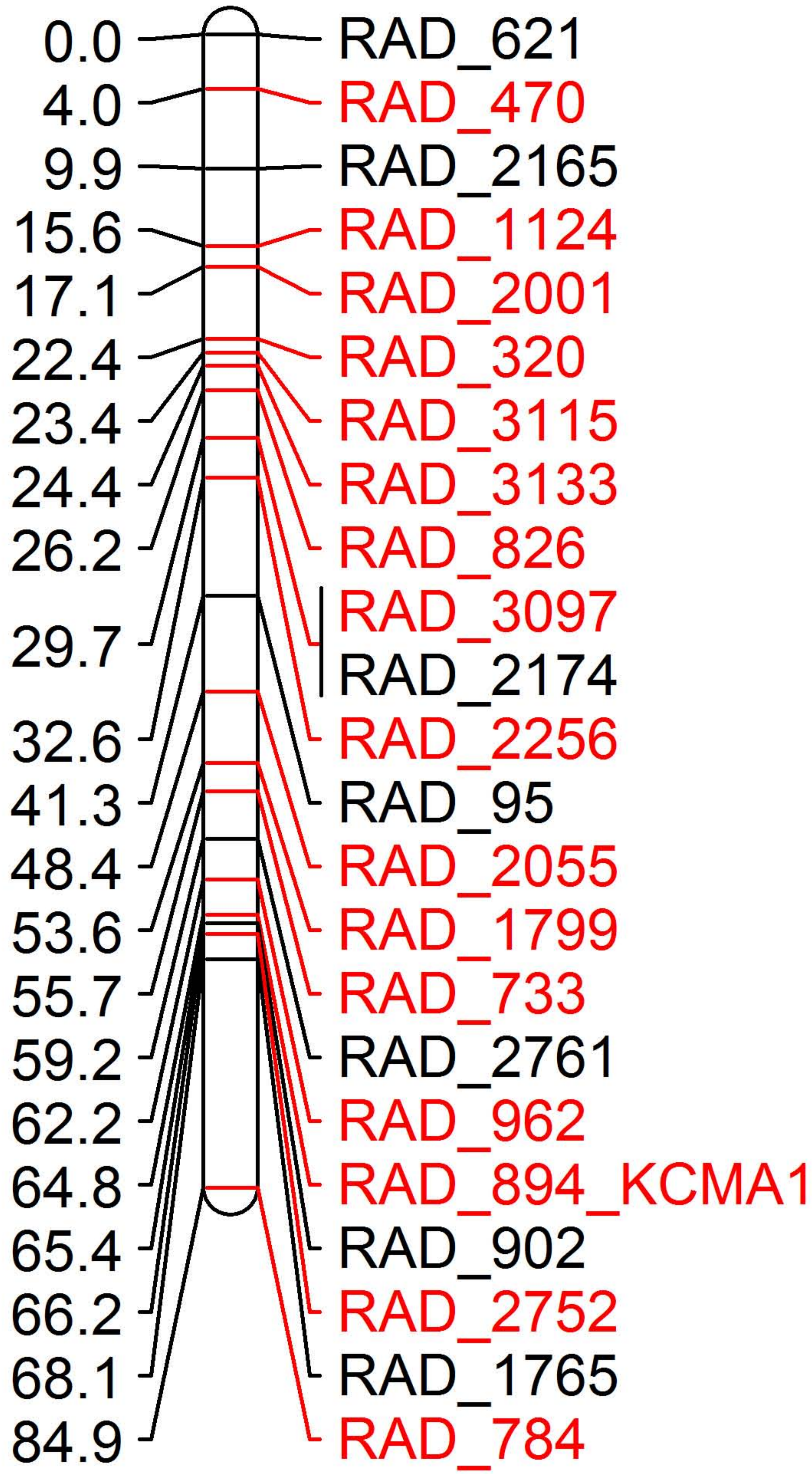

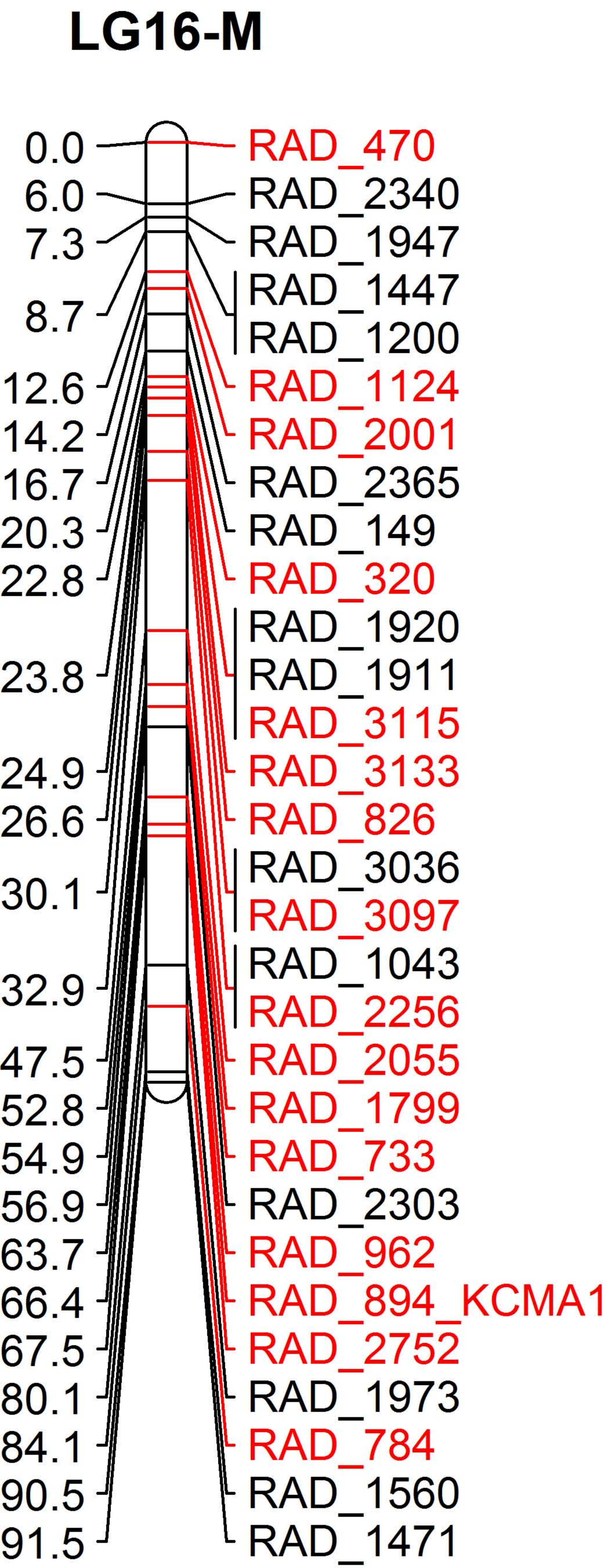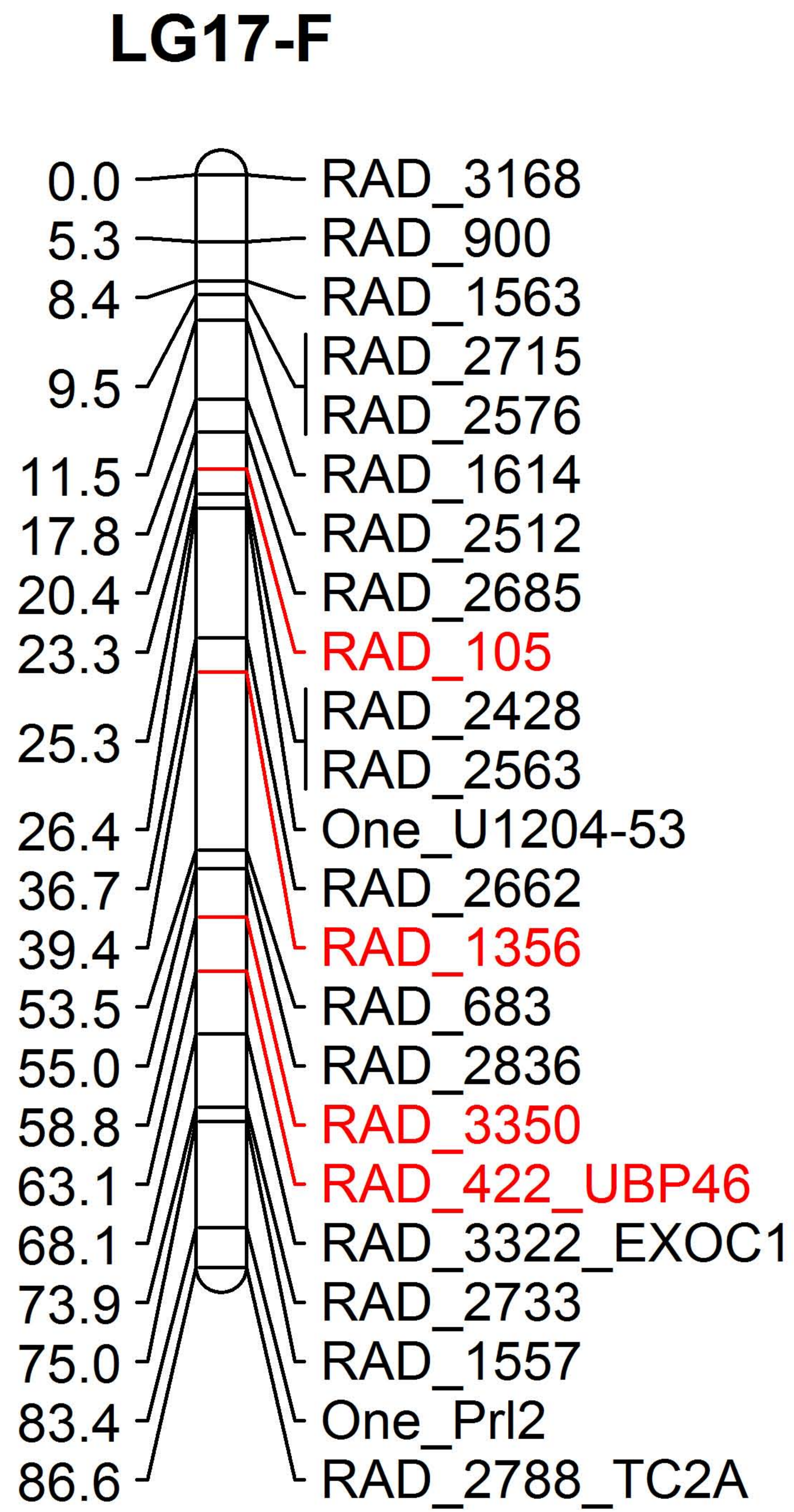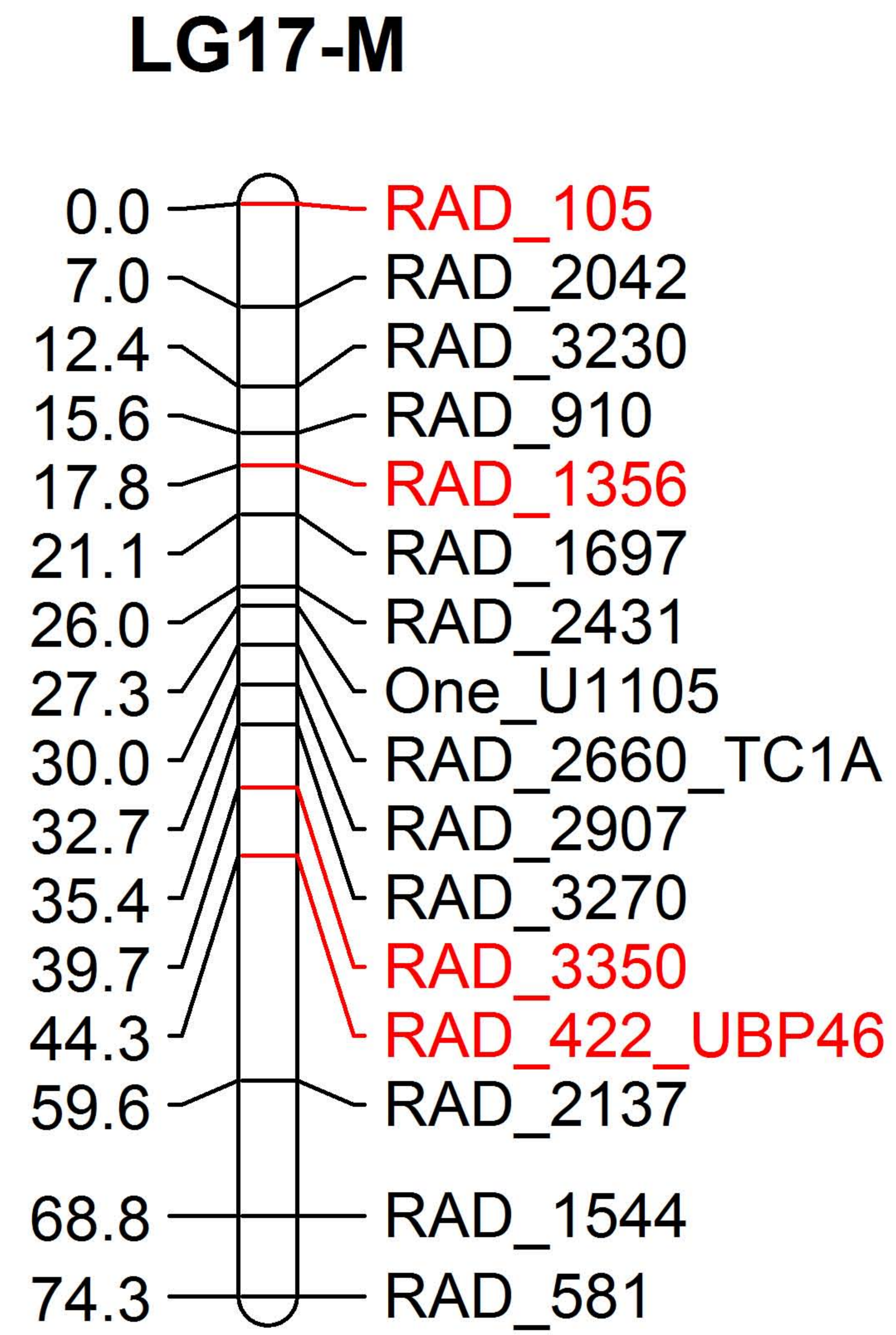

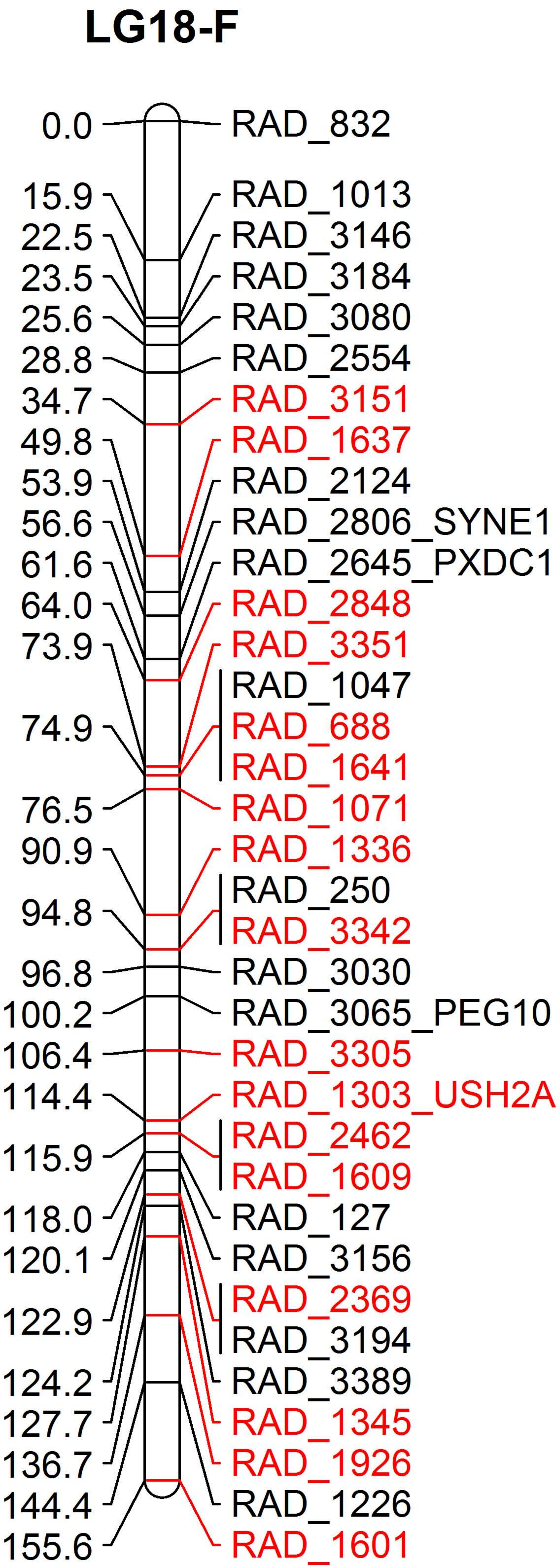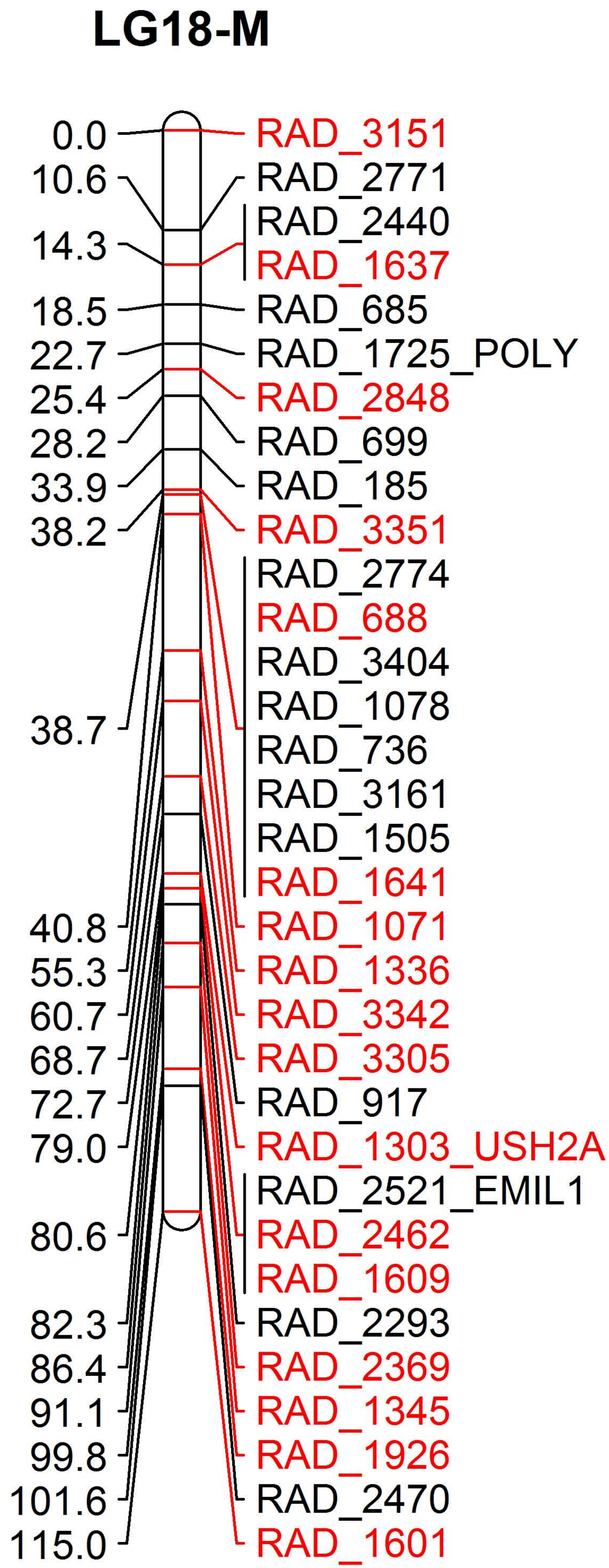

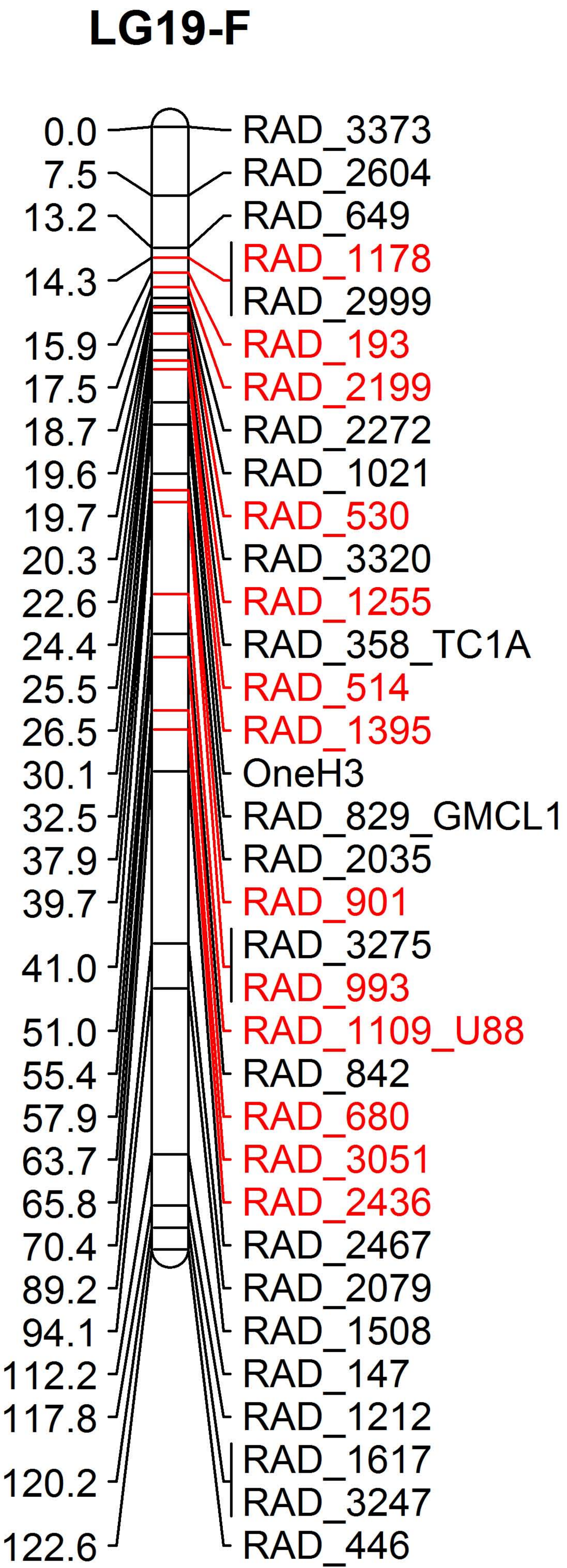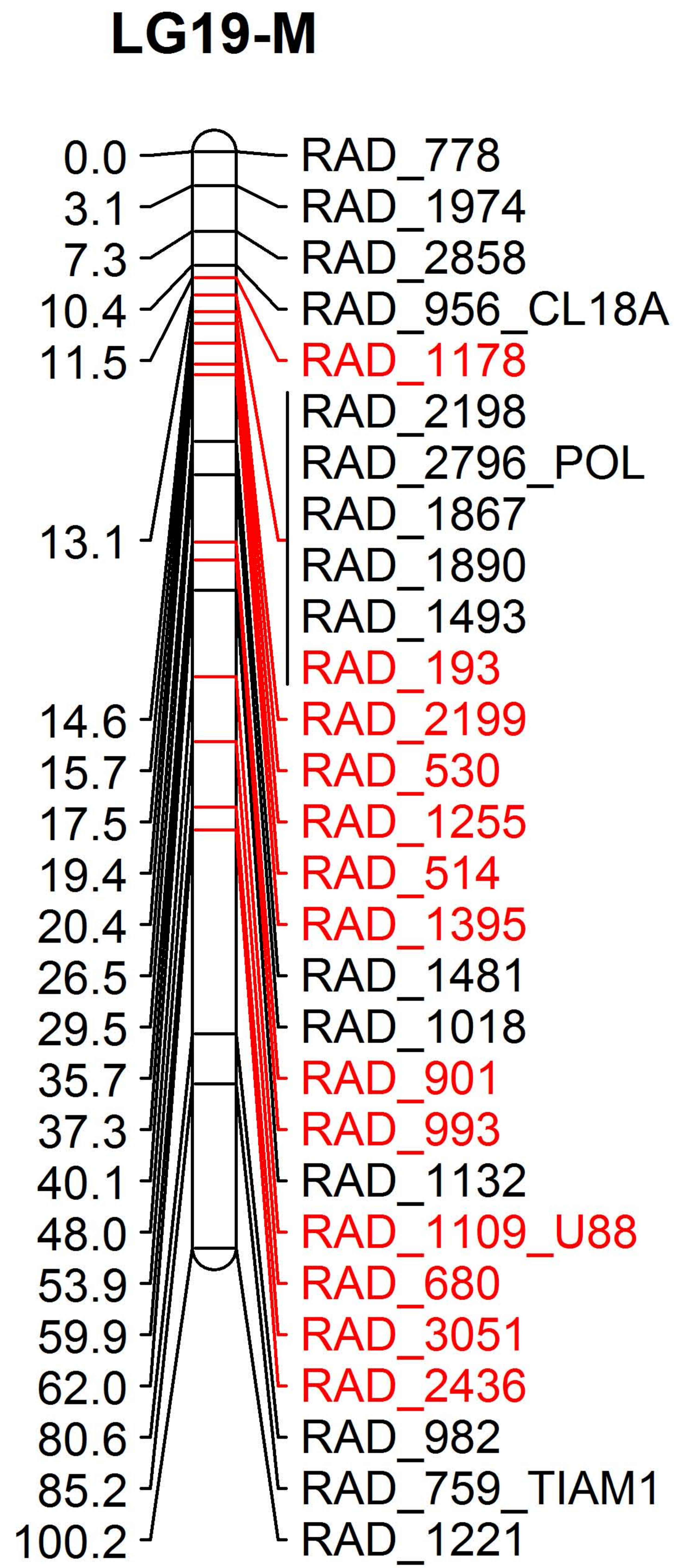

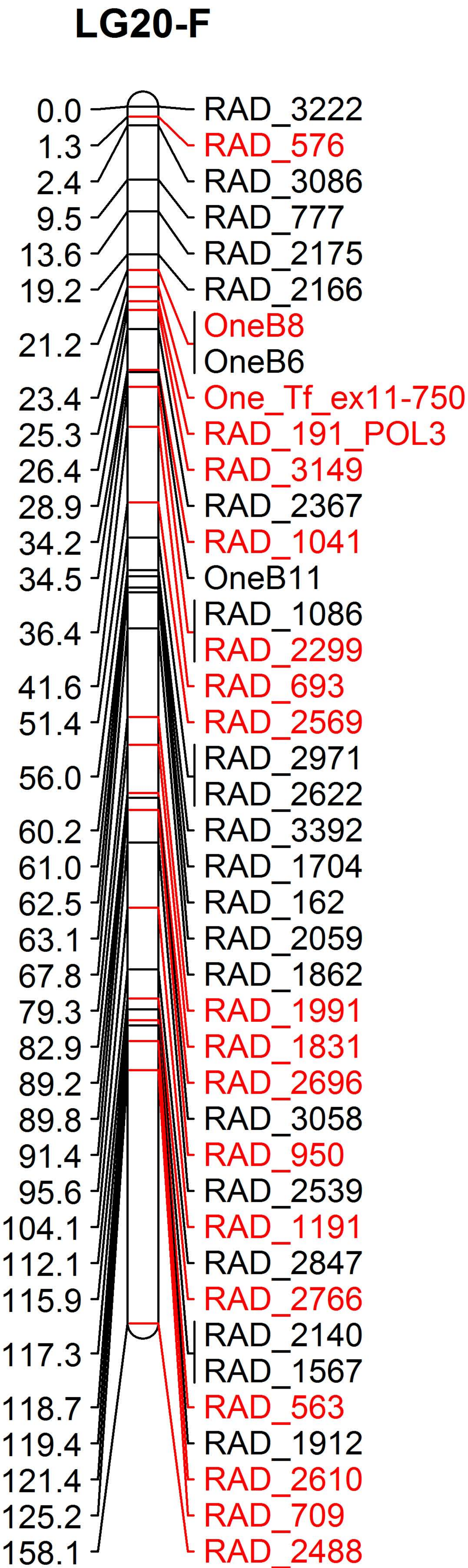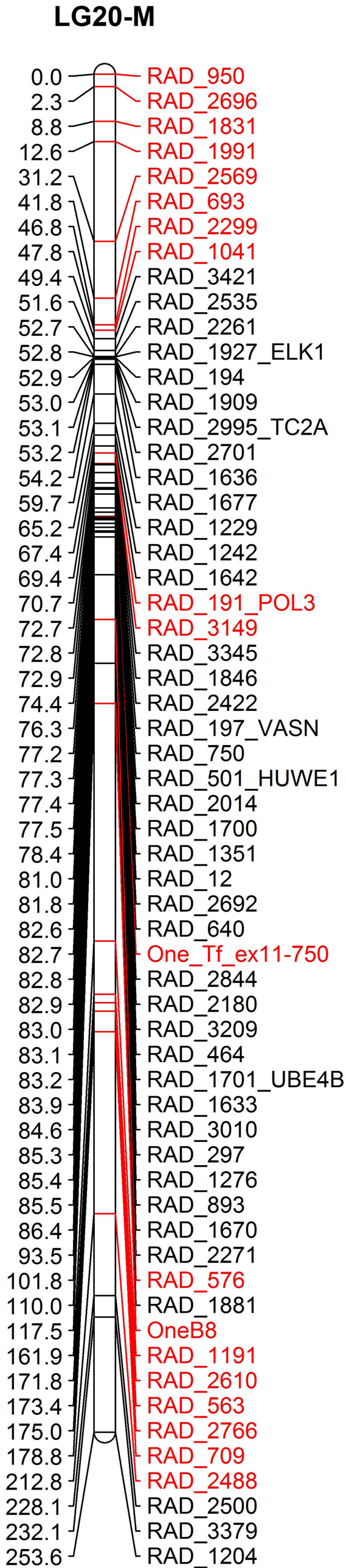

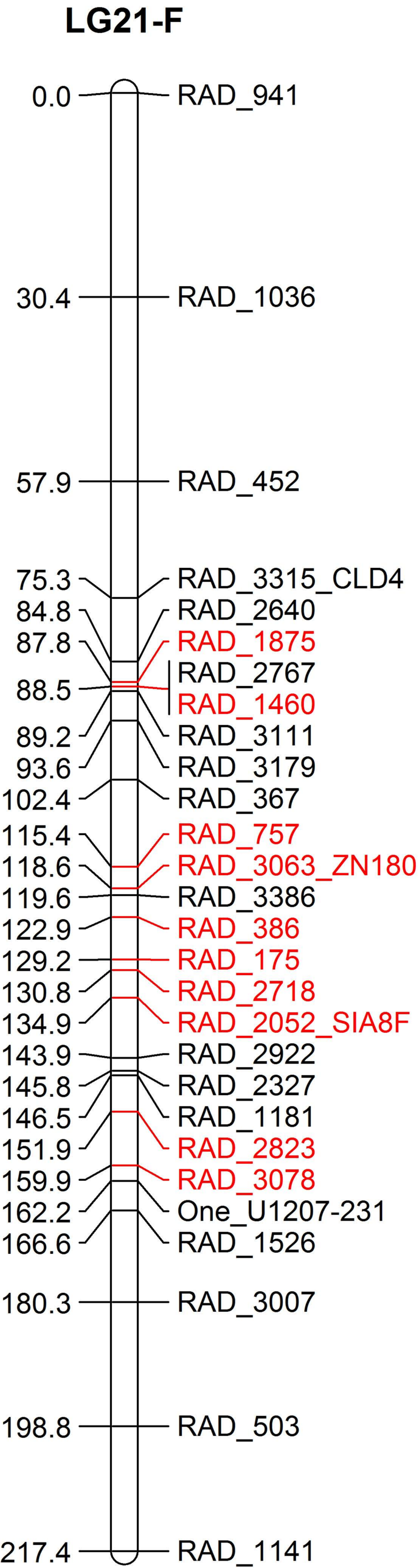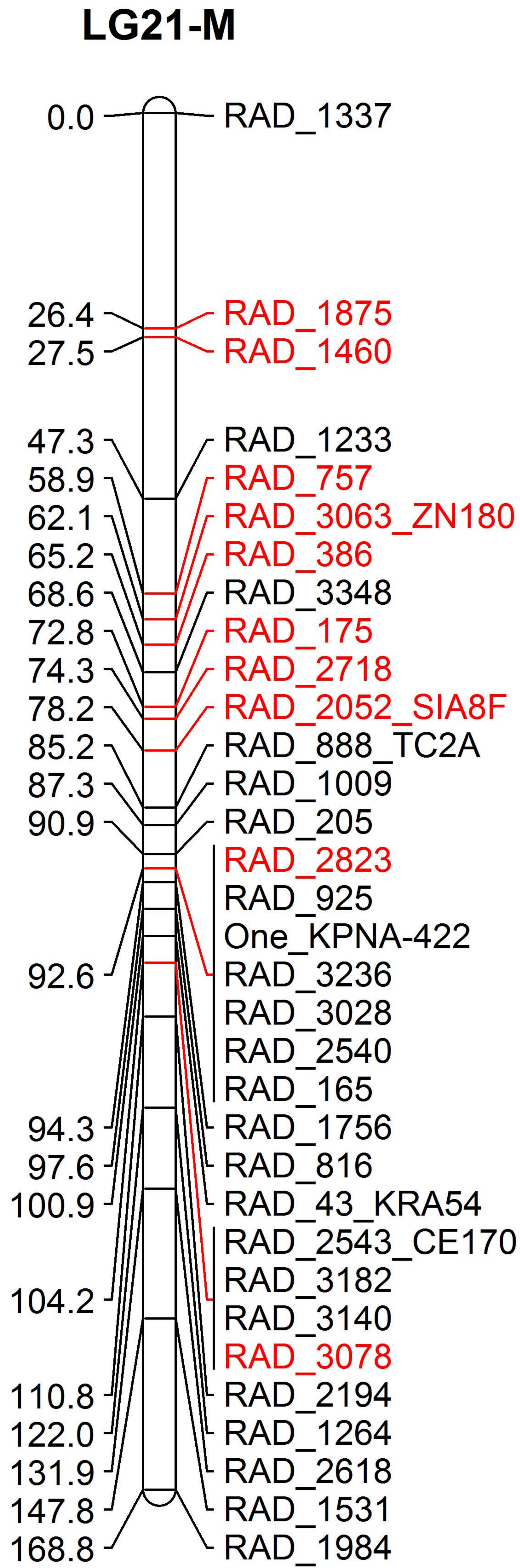

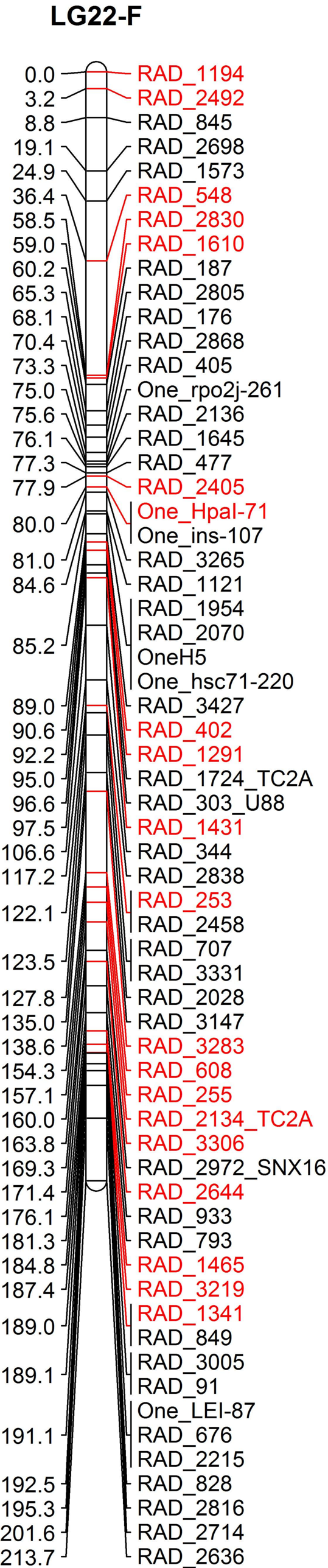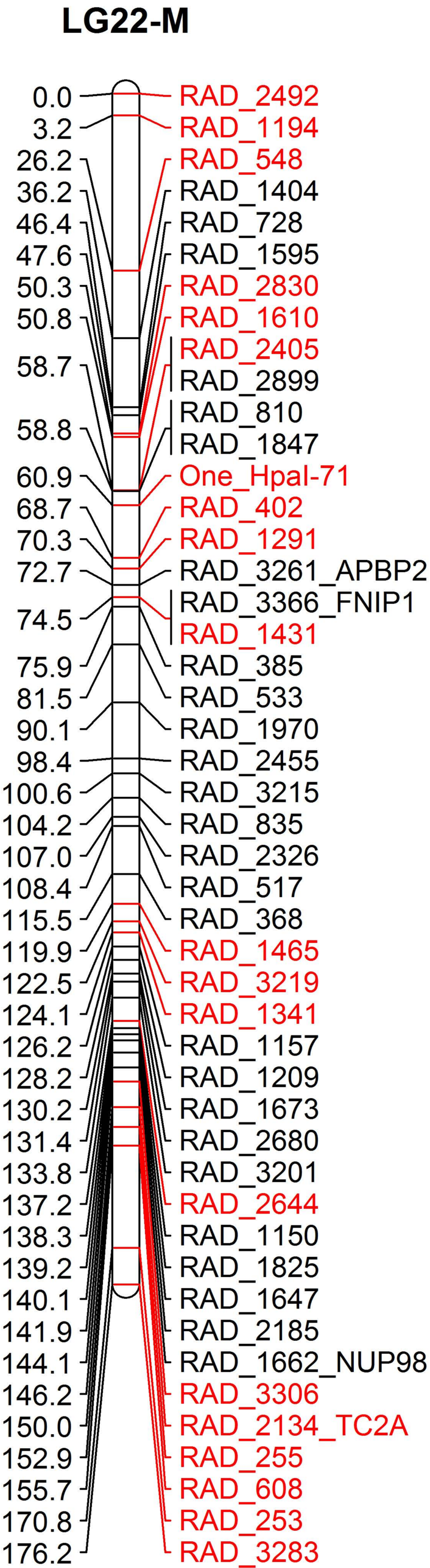

LG23-F

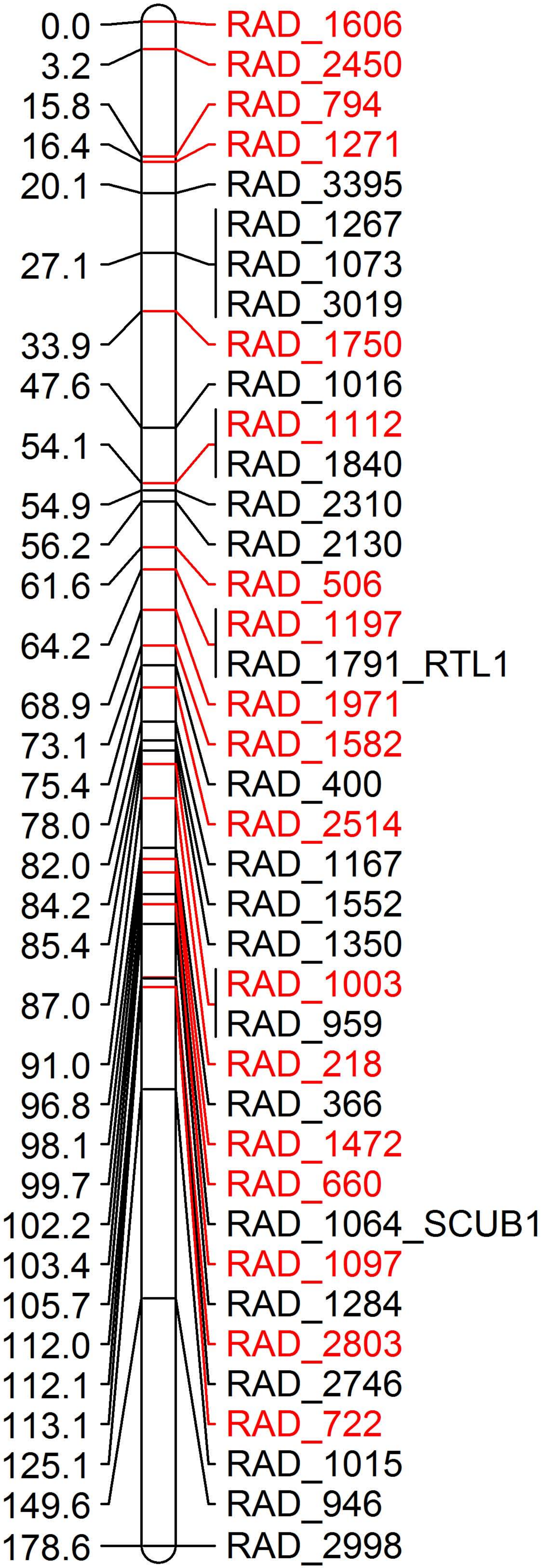

LG23-M

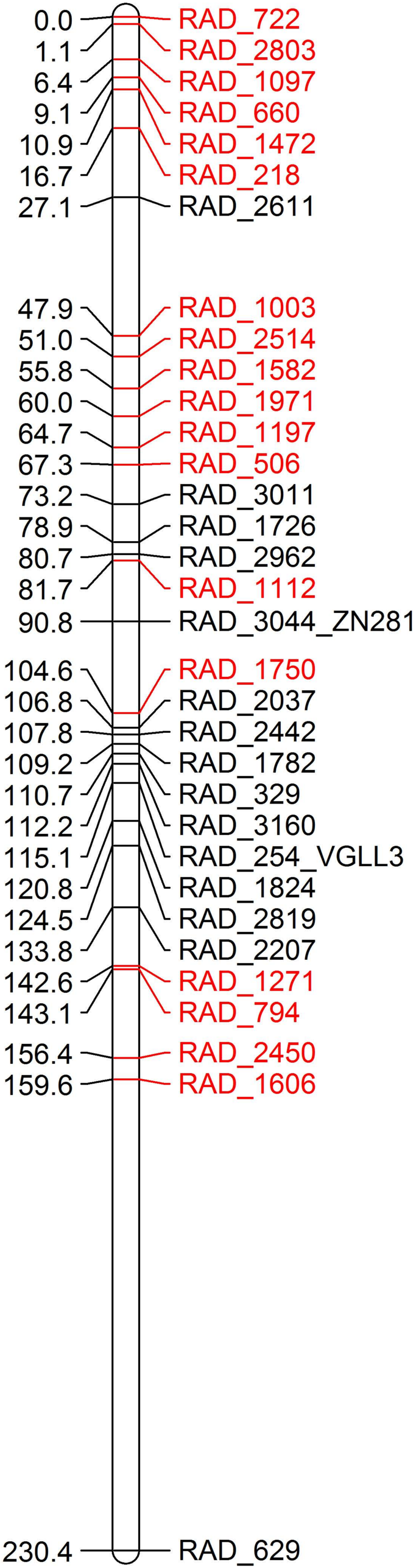

LG24-F

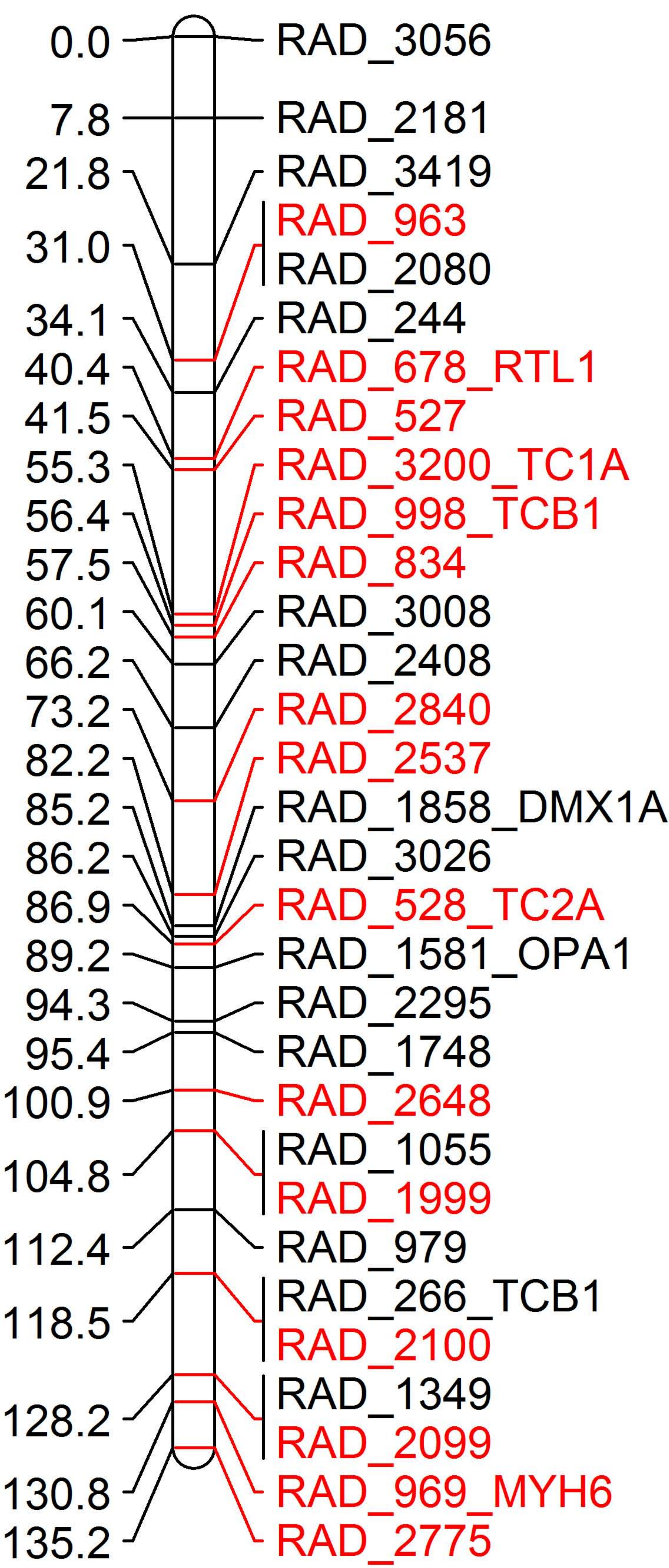

LG24-M

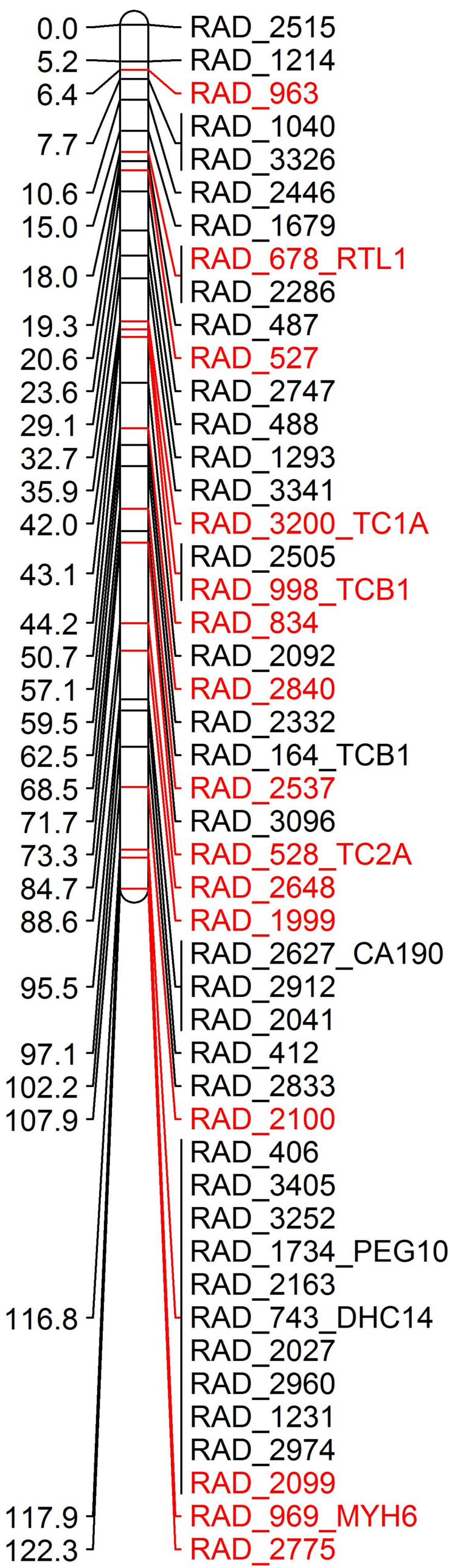

LG25-F

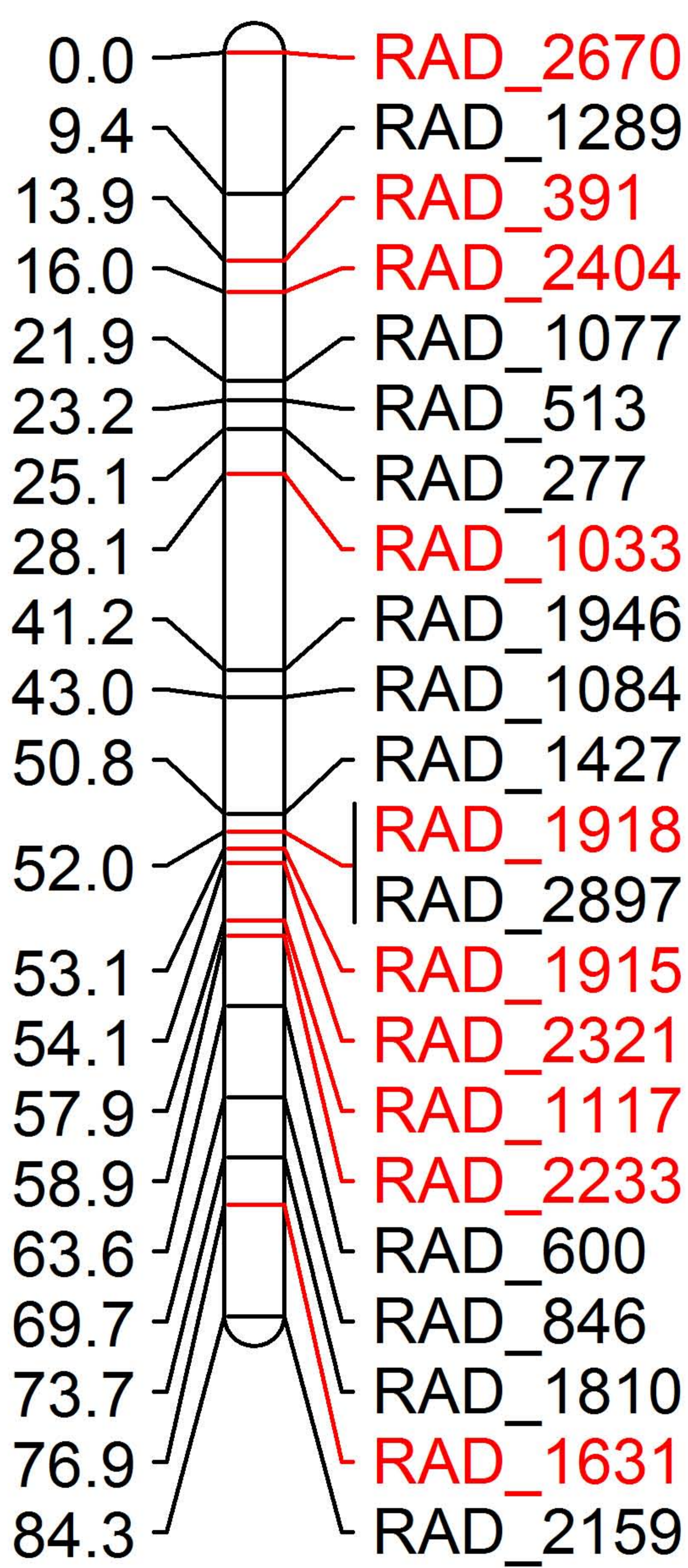

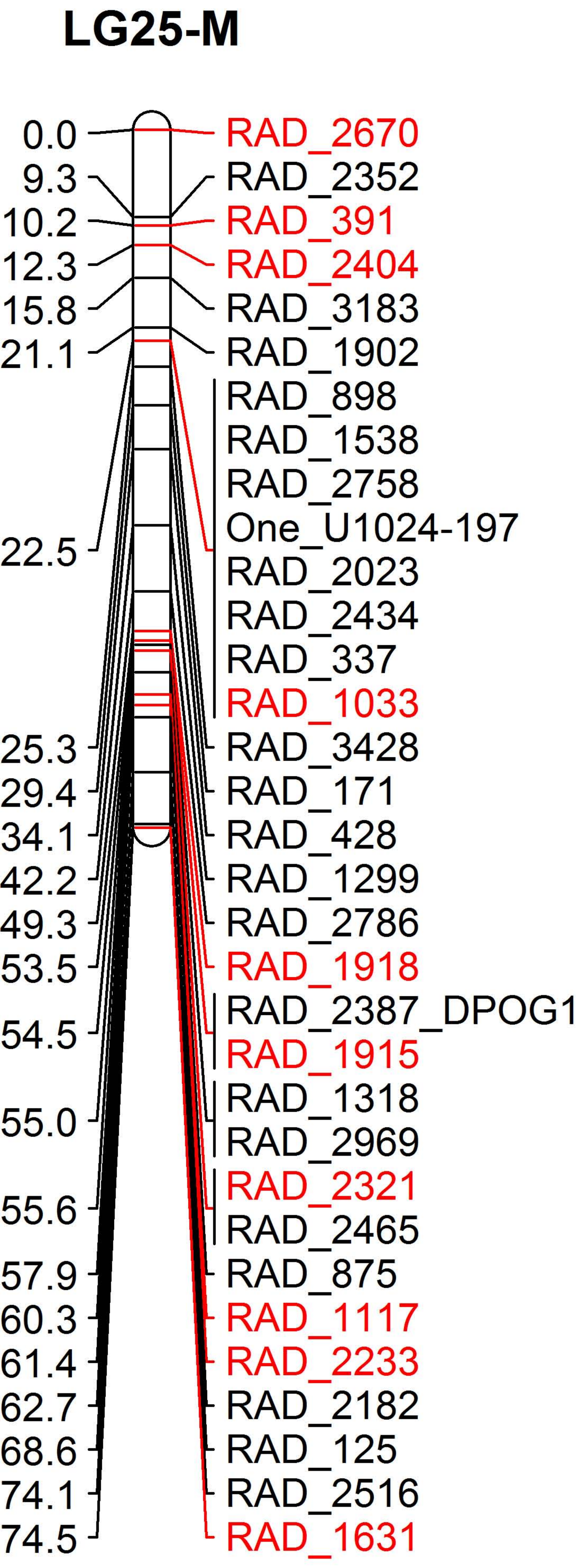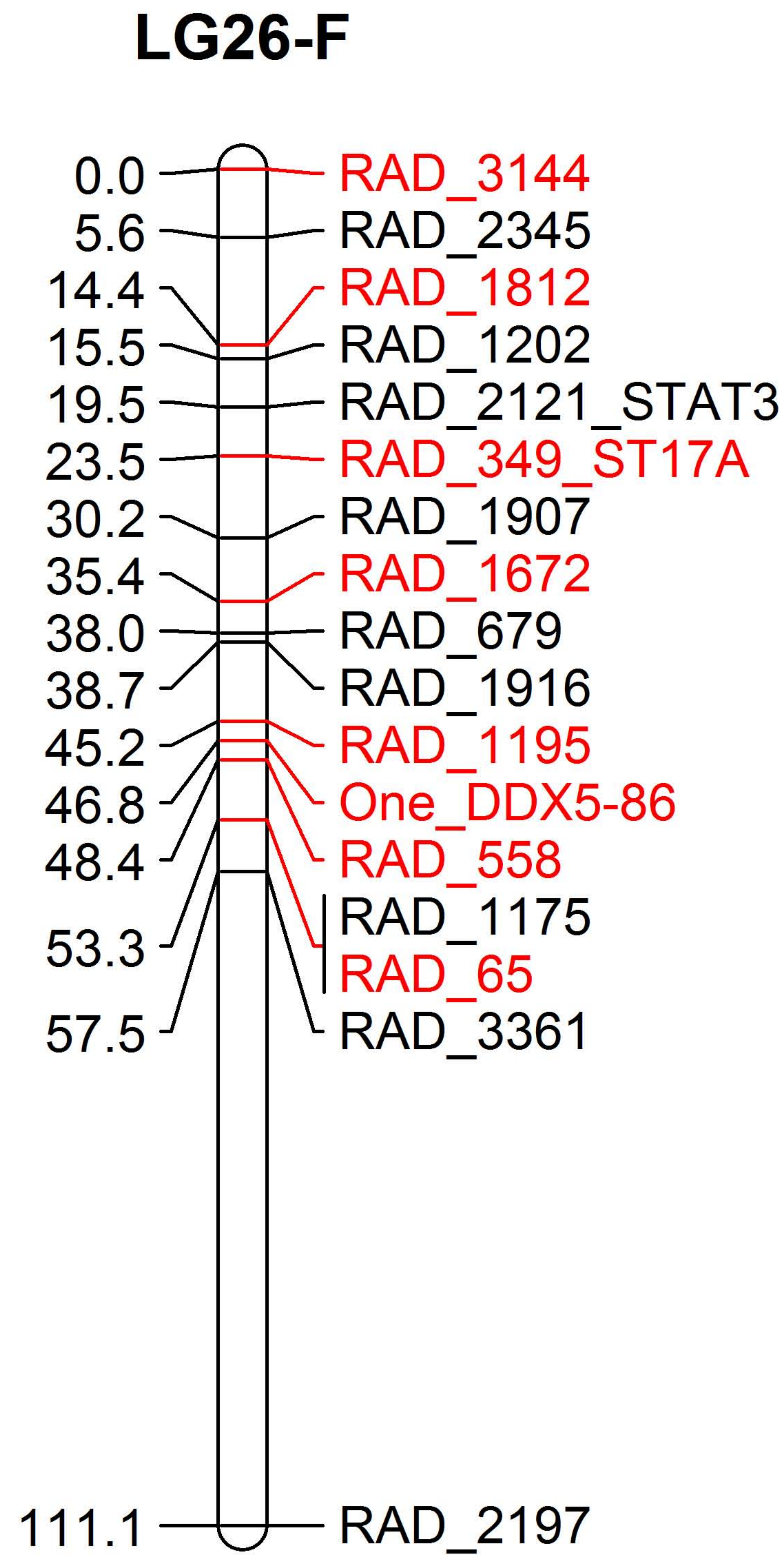

LG26-M

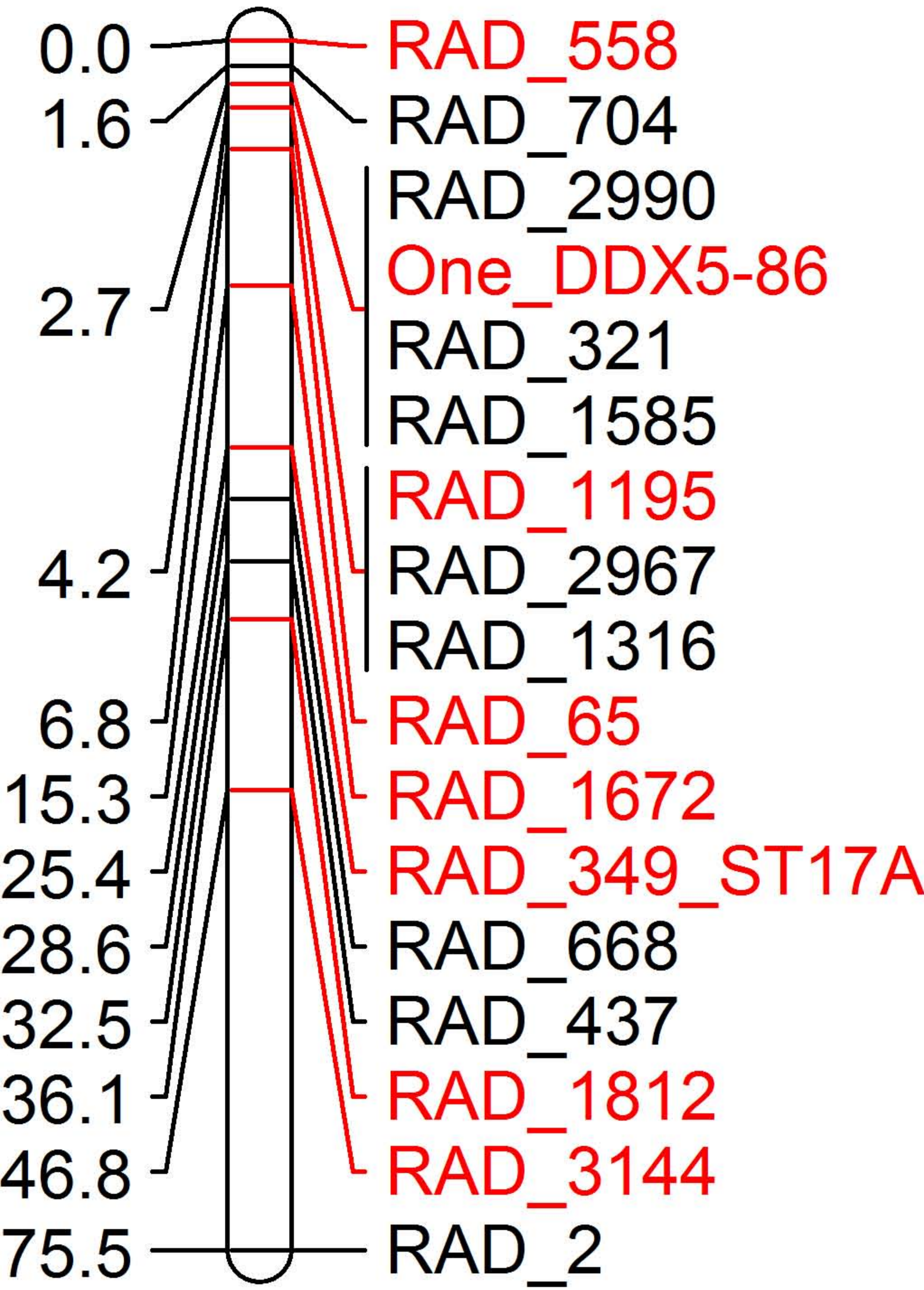

LG27-F

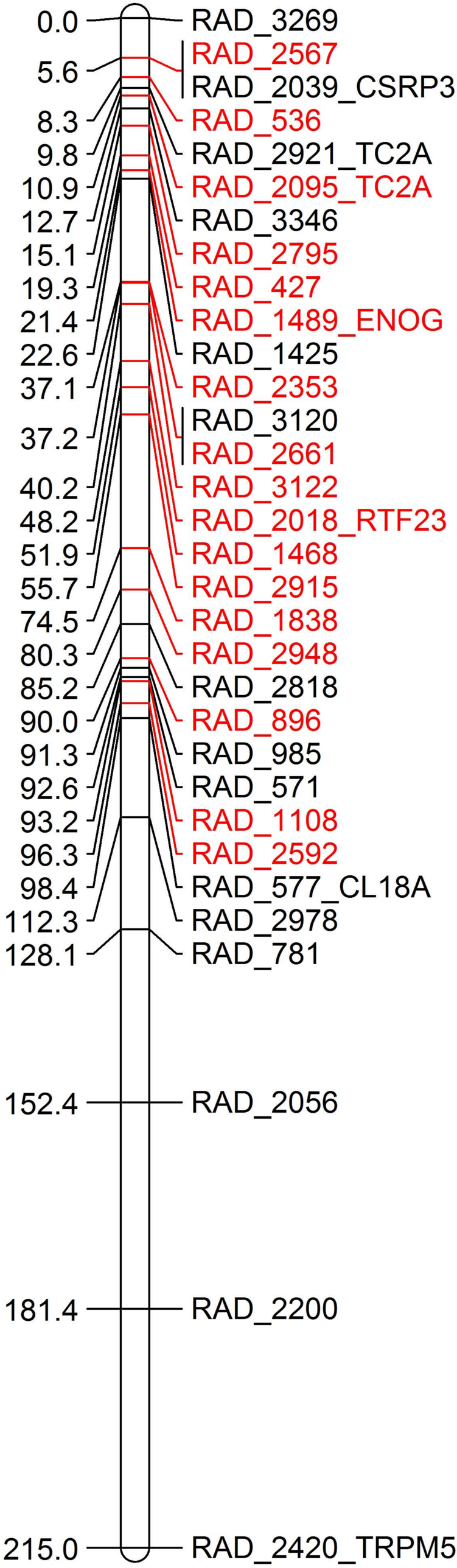

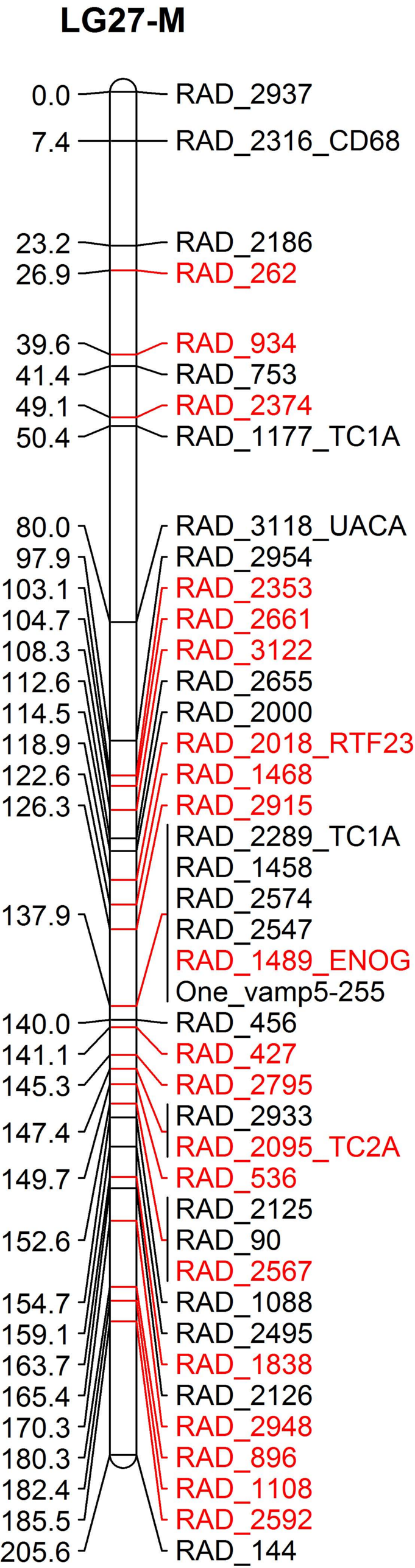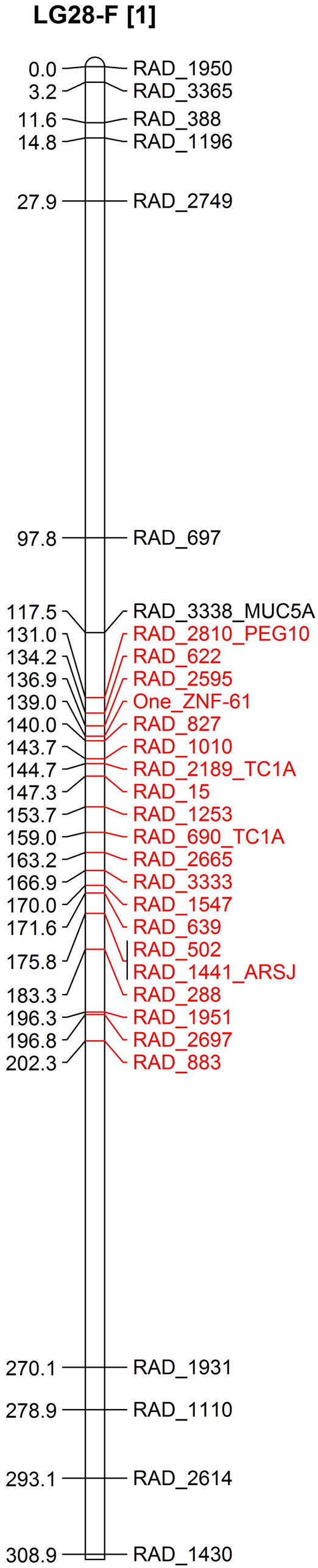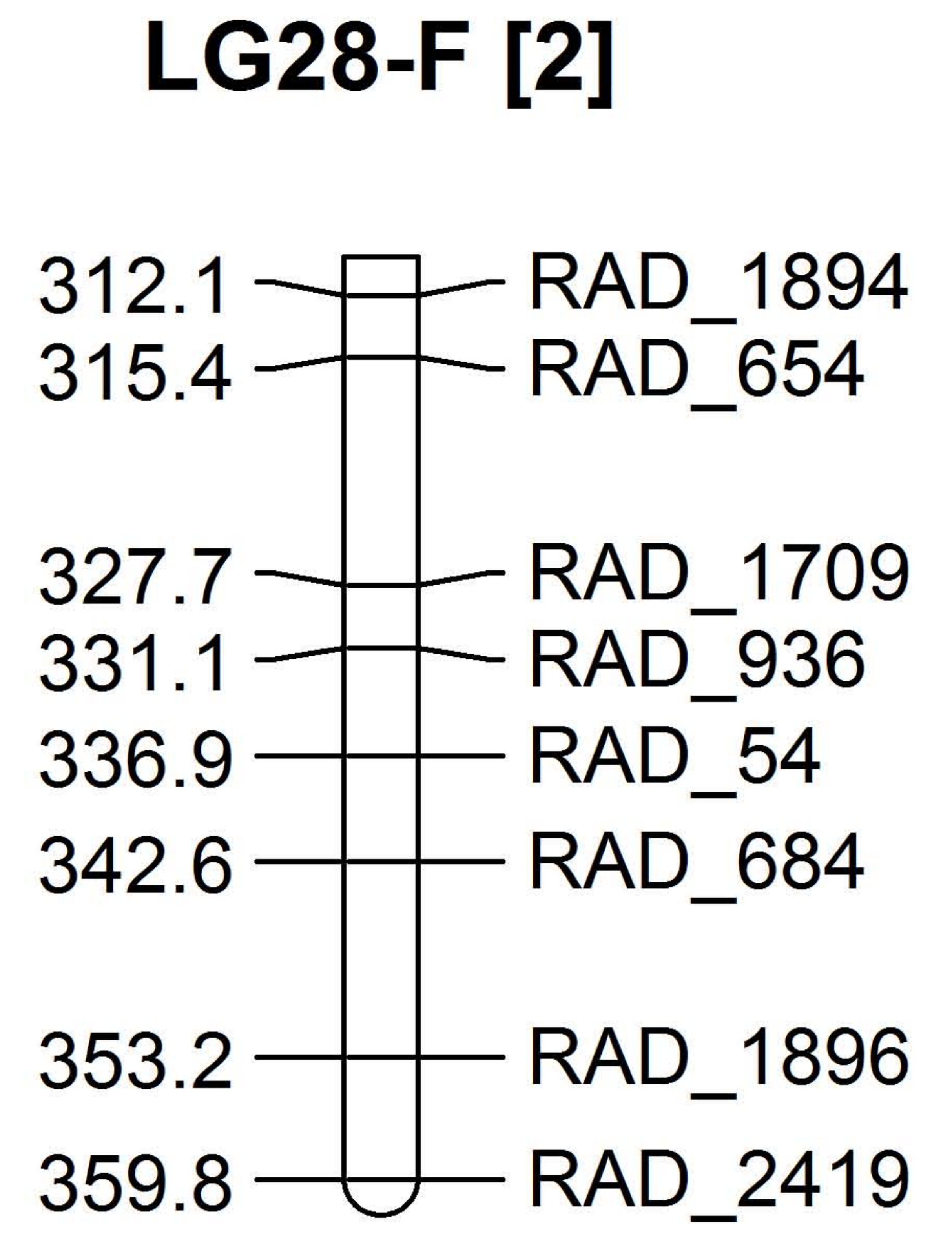

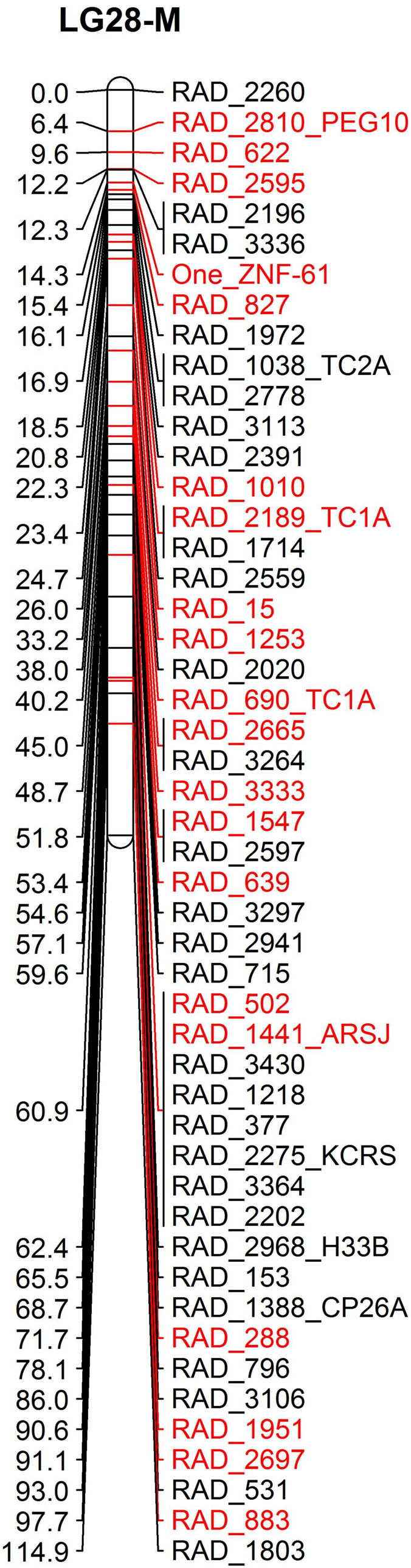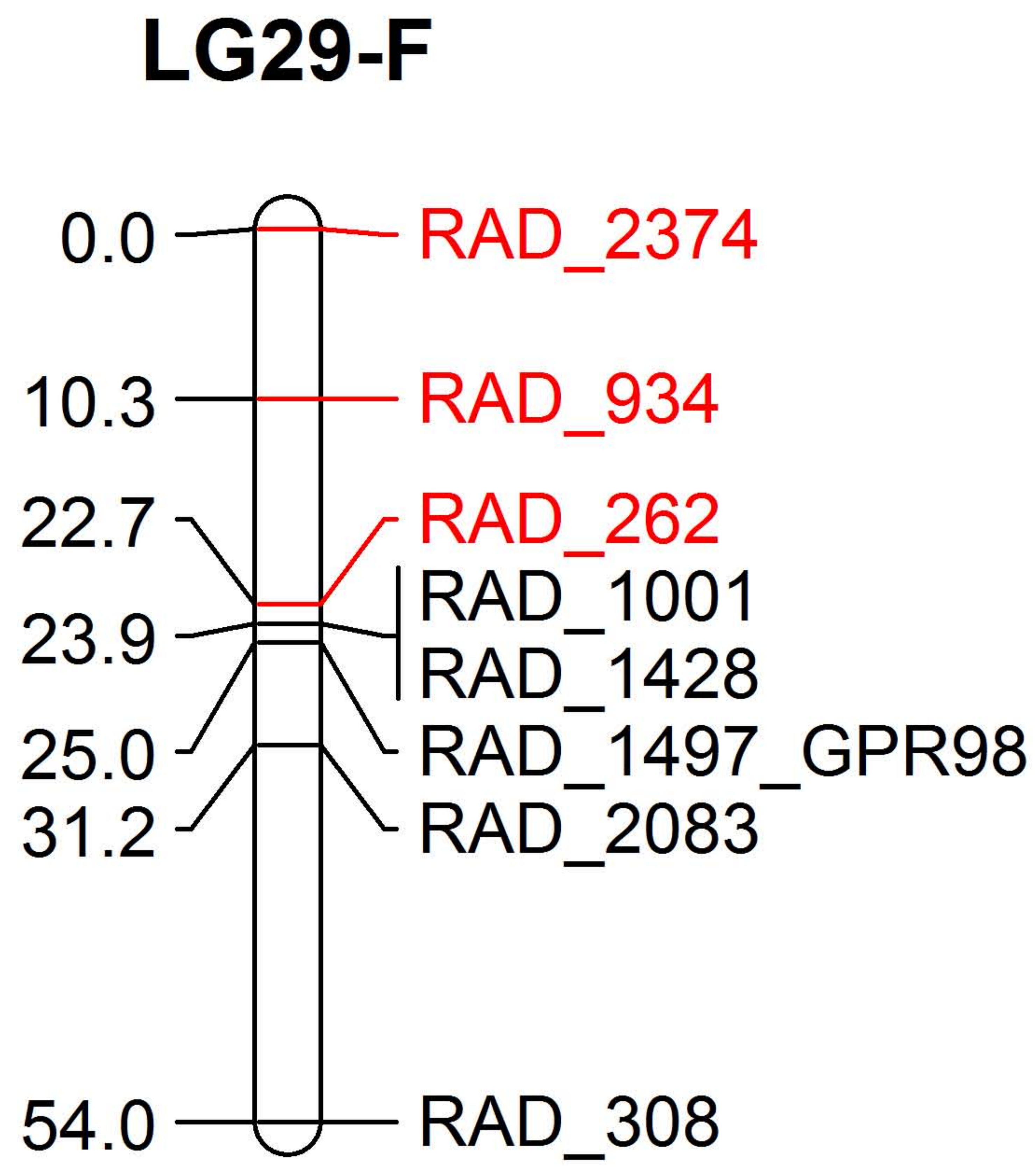

Supplement: Additional file 7 — Supplemental_File_2b. Graphical figures of sockeye salmon meiotic maps. [file 1471-2164-13-521-S7.pdf]
